# The balance between fitness advantages and costs drives adaptation of bacteriophage Qβ to changes in host density at different temperatures

**DOI:** 10.3389/fmicb.2023.1197085

**Published:** 2023-05-25

**Authors:** Mara Laguna-Castro, Alicia Rodríguez-Moreno, Elena Llorente, Ester Lázaro

**Affiliations:** Centro de Astrobiología (CAB), CSIC-INTA, Torrejón de Ardoz, Spain

**Keywords:** bacteriophage Qβ, host density, experimental evolution, fitness cost, adaptation, molecular evolution, temperature

## Abstract

**Introduction:**

Host density is one of the main factors affecting the infective capacity of viruses. When host density is low, it is more difficult for the virus to find a susceptible cell, which increases its probability of being damaged by the physicochemical agents of the environment. Nevertheless, viruses can adapt to variations in host density through different strategies that depend on the particular characteristics of the life cycle of each virus. In a previous work, using the bacteriophage Qβ as an experimental model, we found that when bacterial density was lower than optimal the virus increased its capacity to penetrate into the bacteria through a mutation in the minor capsid protein (A1) that is not described to interact with the cell receptor.

**Results:**

Here we show that the adaptive pathway followed by Qβ in the face of similar variations in host density depends on environmental temperature. When the value for this parameter is lower than optimal (30°C), the mutation selected is the same as at the optimal temperature (37°C). However, when temperature increases to 43°C, the mutation selected is located in a different protein (A2), which is involved both in the interaction with the cell receptor and in the process of viral progeny release. The new mutation increases the entry of the phage into the bacteria at the three temperatures assayed. However, it also considerably increases the latent period at 30 and 37°C, which is probably the reason why it is not selected at these temperatures.

**Conclusion:**

The conclusion is that the adaptive strategies followed by bacteriophage Qβ, and probably other viruses, in the face of variations in host density depend not only on their advantages at this selective pressure, but also on the fitness costs that particular mutations may present in function of the rest of environmental parameters that influence viral replication and stability.

## 1. Introduction

The infection cycle of any virus can be divided into two phases, an intracellular one, corresponding to the period of replication inside the cell, and an extracellular one, comprising the time between infections. In the extracellular phase, viruses behave as inert entities –the virions– that are exposed to the physicochemical conditions of the environment, which can damage the virus particles and compromise the ability to initiate new infections. The time that a virus remains in the extracellular environment is strongly conditioned by virus and host concentration. Due to mass action kinetics, host concentration is the most relevant factor influencing the number of infections that can be initiated from a viral population of a given size (Shao and Wang, 2008). Viruses can select strategies to adapt to low host availability, which usually involve modification of some of the parameters that define their infection cycle. In the case of lytic bacteriophages, these parameters are adsorption rate, duration of the latent period, burst size, and stability in the external medium (Hyman and Abedon, 2009; Bull and Gill, 2014; Dennehy and Abedon, 2020a,b). The adsorption rate is the ability of a phage to interact with the cell receptor in order to introduce its genome into the bacterium. The latent period is the time that elapses from the time a phage genome enters the cell until a viral progeny is released. The burst size is the number of phages produced per infected bacterium. Finally, stability in the extracellular medium is other relevant factor influencing the time that a virion remains infective. The optimal combination of values for all these parameters is not fixed, but depends on the environmental conditions to which each particular phage population is exposed (Woody and Cliver, 1995; Chantranupong and Heineman, 2012; Inomata et al., 2012; Yin and Redovich, 2018).

Sometimes, improving one of the parameters described above implies worsening others, that is, it has a fitness cost (Shao and Wang, 2008; Chantranupong and Heineman, 2012; Goldhill and Turner, 2014). An example is the increase in extracellular stability, which is sometimes associated with a decrease in the ability to infect (Elena, 2001; De Paepe and Taddei, 2006; Wasik et al., 2015). Other times, a parameter can only be optimized within a range of values that, when exceeded above or below, has negative consequences. For instance, increasing the adsorption rate may be favorable when there are few bacteria available, but, if these are very scarce, they could be exhausted very quickly, resulting in an infective process of very short duration. Increasing the latent period causes infections to progress more slowly, allowing a fraction of bacteria to continue to reproduce so that they can be infected in subsequent cycles (Abedon et al., 2001, 2003). This strategy has an extra value when conditions in the external environment are very hostile and, therefore, phages would spend less time exposed to them. However, if the latent period is too long, the number of infection rounds in a given period of time will also be lower (Heineman and Bull, 2007), which can reduce the final virus yield. Even it might happen that, when the viral progeny is released, bacteria have reached a state in which they are less susceptible to infection (Abedon, 2016). Finally, variations in burst size are not easy to interpret. Although, in principle, increases in this parameter might seem positive, they usually occur at the cost of increasing the latent period (Abedon et al., 2003), which, as we have indicated, is not always favorable. Habitat structure can also modulate the effect of the increase of the burst size (Dennehy et al., 2007). The conclusion is that the adaptive strategy that a population of bacteriophages will follow when bacteria availability is limited is not easy to predict and will depend on the whole combination of conditions of the extracellular and intracellular environment.

In order to study the changes in the infective cycle of phages in response to host density variation, we used bacteriophage Qβ as an experimental model. Qβ is a lytic phage that infects the bacterium *Escherichia coli* using as receptor the conjugative F pilus. It is a member of the *Leviviridae* family (genus Allolevivirus) (Olsthoorn and Van Duin, 2011). It has a single-stranded, positive-sense RNA genome of 4217 nucleotides that encodes four proteins: A2 or maturation protein, which is present in a single copy and mediates phage binding to the bacterial pili, penetration of the viral genome, and cell lysis; coat protein, which is the major capsid protein; A1 or the minor capsid protein, which is produced occasionally when the stop codon of the capsid protein is read as tryptophan; and the replicase that copies the RNA genome. The precise function of the A1 protein is currently unknown, although it is essential for infection to occur (Hofstetter et al., 1974). It consists of the full-length coat protein connected by a flexible linker to an extension of 196 amino acids at the C-terminal end (Rumnieks and Tars, 2011) that is located on the exterior of the capsid (Vasiljeva et al., 1998). Around 10 copies of A1 are present in the capsid, replacing monomers of the coat protein. Regarding the entry mechanism of Qβ in its host, studies carried out with the related phage MS2, which also infects *E. coli* through the pilus, indicate that, after binding of the phages, continued pilus retraction brings them close to cell surface, where the complex formed by the Mat protein (which interacts with the pilus in MS2) and the virus genomic RNA penetrates inside the central channel that traverse the plasma membrane at the basis of the pilus and is driven into the host cytosol (Dent et al., 2013; Meng et al., 2019). Due to the similarities between MS2 and Qβ (Gorzelnik and Zhang, 2021), a similar mechanism probably is also working in the latter. During the process, a torsional stress is produced that causes the detachment of the pilus and its release to the environment, which has been demonstrated for both phages (Harb et al., 2020).

In a previous work, we studied the adaptation of bacteriophage Qβ to replicate in the presence of different bacterial densities at the optimal replication temperature for the virus (37°C) (Laguna-Castro and Lázaro, 2022). A fraction of the progeny produced after a 2-h incubation was used to initiate a new infection using fresh bacteria at the same concentration as in the previous culture, a process that was repeated for 16 serial transfers. This experimental design prevents evolution of bacteria and permits a reset at each transfer of the selective pressure that we are studying (the bacterial density in this case). The results we obtained showed that in all populations that had evolved at a bacterial density below a given value, a mutation was selected (C2011A), which produces the amino acid change T222N in the A1 protein. The main effect of the mutation was to increase the entry of the virus into the bacterium. The latent period and the stability of the phage in the extracellular medium were not affected, but there did seem to be a decrease in the burst size. These findings suggest that A1 protein could contribute to the initial binding of Qβ to the F-pilus, increasing in this way phage-bacteria interaction.

In the experiments presented in this work, we have studied the effect of varying cell density at the same time that another essential parameter for virus replication, temperature, is also modified. Temperature largely influences the tertiary structure of proteins. Therefore, it is to be expected that all processes involving interaction between molecules and structures (for instance between phage and bacteria components) or enzymatic catalysis are affected by variations in this parameter. The impact of temperature changes on Qβ replication and evolution has been previously studied in depth in our laboratory, although always at the same bacterial density that we use as a standard. While the decrease in temperature (from 37 to 30°C) does not strongly decrease viral titers, the increase (from 37 to 43°C) produces drastic reductions in the virus yield (Arribas et al., 2014). Nevertheless, the phage is able to adapt to the latter condition through mutations in all its genes (Arribas et al., 2014, 2016; Kashiwagi et al., 2014; Lázaro et al., 2018; Somovilla et al., 2019, 2022; Arribas and Lázaro, 2021). We thought that our experience in Qβ adaptation to 30 and 43°C might be very useful in differentiating the mutations that are selected in response to each of the two main selective pressures that are acting in these new experiments (change in temperature and host density). Therefore, we chose 30 and 43°C to study whether adaptation of Qβ to variations in host density was temperature-dependent.

The new results we have obtained show that, when bacteria availability is reduced, bacteriophage Qβ selects a common adaptive strategy both at 30 and 43°C, which is based in the improvement of its entry into bacteria. However, while at 30°C adaptation is achieved through the same mutation in the A1 protein that was selected at 37°C, at 43°C it does so through a different mutation located in the A2 protein (V256A), which has strong fitness costs at lower temperatures. It appears, therefore, that adaptation of bacteriophage Qβ to low host availability involves different mutational pathways depending on the balance between fitness advantages and costs at each replication temperature.

## 2. Materials and methods

### 2.1. Viruses and bacteria. Standard procedures for infection

The plasmid pBRT7Qβ, which contains a cDNA of bacteriophage Qβ cloned in the plasmid pBR322 (Taniguchi et al., 1978; Barrera et al., 1993), was used to transform *Escherichia coli* DH5-α, a strain that permits virus expression, although it cannot be infected because it lacks the virus receptor. The supernatant of an overnight culture, obtained from a transformed colony, was used to infect *E. coli*, strain Hfr (Hayes, 1953), in semisolid agar. The virus progeny contained in a randomly chosen lysis plaque was isolated, and 10^6^ plaque forming units (pfu) were used to infect an *E. coli* Hfr culture under standard conditions (37°C, 250 rpm) for 2 h. The supernatant of this culture was used as the ancestor of all the evolutionary lineages analyzed in this work. It was denoted Qβ_Anc_ and its consensus sequence showed no mutations relative to the Qβ cDNA cloned in pBR322. The virus Qβ_Anc_ is the same described in our previous work concerning Qβ adaptation to low host density (Laguna-Castro and Lázaro, 2022).

Qβ was propagated in *E. coli* Hfr in NB medium (8 g/l Nutrient Broth from Merck and 5 g/l NaCl). Infections in liquid medium were carried out using fresh log-phase *E. coli* cultures with an OD_600_ of 0.8 that were infected with the amount of pfu indicated in each experiment. Cultures were incubated at the indicated temperature for 2 h with good aeration (250 rpm). To estimate the total virus yield, cultures were treated either with chloroform (1/20 v/v, 28°C, 15 min, shaking 850 rpm in thermoblock) or with egg white lysozyme (Sigma-Aldrich; 0.8 mg/ml, 37°C, 30 min, shaking 850 rpm in thermoblock). Virus supernatants were harvested upon centrifugation at 13000 × *g* and maintained at 4°C for short-term use (less than 15 days) or at −80°C for long-term storage. Virus titers were determined by plaque assay and expressed as the number of pfu per ml of the phage suspension.

### 2.2. Evolution experiment

**FIGURE 1:**
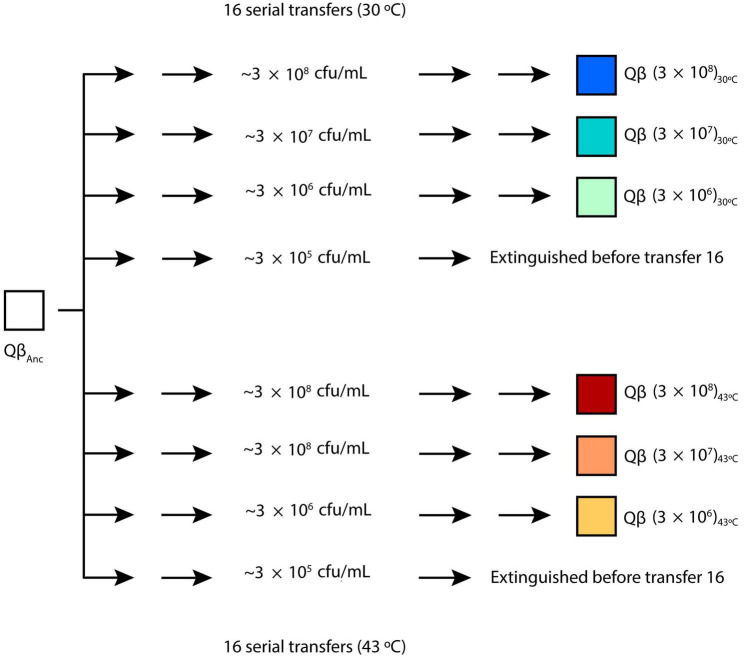
Evolution experiment. The virus Qβ_Anc_ (see section “2.1. Viruses and bacteria. Standard procedures for infection”) was propagated for 16 serial transfers (or until the virus was extinguished), carried out at different bacteria densities either at 30 or 43°C. Evolutionary lineages obtained at transfer number 16 were denoted Qβ (3 × 10^8^)_30°C_, Qβ (3 × 10^8^)_43°C_, Qβ (3 × 10^7^)_30°C_, Qβ (3 × 10^7^)_43°C_, Qβ (3 × 10^6^)_30°C_, and Qβ (3 × 10^6^)_43°C_ to indicate the bacterial density (expressed in cfu/ml and shown in brackets) and the temperature used at each transfer. Lineages propagated at the bacterial density of 3 × 10^5^cfu/ml were extinguished before transfer 16. Two replicas were performed for each condition. When necessary, the numbers 1 and 2 were used to distinguish them. For additional details, see the section “2.2. Evolution experiment”.

The virus Qβ_Anc_ was used to initiate 8 replicate evolutionary lineages differing in the bacterial density (Figure 1). Propagation took place for 16 serial transfers (or until the virus was extinguished) either at 30 or 43°C. At each transfer, bacteria were freshly prepared by growing *E. coli* at 37°C until an OD_600_ of 0.8, which according our estimations corresponds to 6 × 10^8^ colony forming units (cfu) per ml.^1^ The culture was serially diluted in NB medium (10^–1^, 10^–2^, and 10^–3^ dilutions), and 0.5 ml of either the undiluted culture or the corresponding dilution were placed in 10 ml tubes containing 400 μL of NB. Infections were initiated with 10^7^ pfu in 100 μL of phage buffer (25 mM Tris–HCl pH 7.5, 5 mM MgCl_2_, 0.5 g/l gelatin). After 2 h of incubation under standard conditions either at 30 or 43°C, the virus supernatants were collected upon treatment with chloroform, and a fraction of the phage suspension (10^7^ pfu or, when the titers did not allow it, the amount of phage contained in 100 μL of the previous supernatant) was used to infect fresh *E. coli* cultures prepared as described above. Evolutionary lineages were denoted Qβ (3 × 10^8^)_30°C_, Qβ (3 × 10^8^)_43°C_, Qβ (3 × 10^7^)_30°C_, Qβ (3 × 10^7^)_43 ^° C_, Qβ (3 × 10^6^)_30°C_, and Qβ (3 × 10^6^)_43°C_ to indicate the bacterial density (in brackets) and the temperature used in the experiment. Lineages propagated at the bacterial density of 3 × 10^5^ cfu/ml were extinguished before transfer 16 and were not used for further experiments. When necessary, the numbers 1 and 2 were used to distinguish the two replicas performed for each condition.

A negative control in which undiluted bacteria were incubated in culture medium in the absence of virus was set at each transfer. This control was processed and plated exactly the same as the experimental samples. When lysis plaques appeared, the corresponding transfer was discarded and repeated again.

Additional evolution experiments using a similar protocol to that described above were performed with single mutant viruses containing mutation U830C (virus Qβ_U830C_), and C2011A (virus Qβ_C2011A_). The replication temperatures and the bacterial densities used are indicated in the text and in the legend of the corresponding figures.

### 2.3. Determination of the virus replicative ability

The virus yield obtained in replication assays carried out in liquid medium was used as a measure of the virus replicative ability. Triplicate liquid cultures containing the indicated bacterial density were inoculated with 10^4^ pfu of the virus population assayed, in a final volume of 1 ml. We used this amount of virus to ensure that the replicative capacity of the system was not saturated. After 2 h of incubation at either 30, 37, or 43°C, the total virus produced was estimated through treatment with 0.8 mg/ml of egg white lysozyme (Sigma-Aldrich) for 30 min at 37°C with shaking (850 rpm in thermoblock). Lysozyme was used instead of chloroform because of its higher efficiency to lysate cells, demonstrated in several lysis assays carried out in our lab (data not shown).

### 2.4. Site-directed mutagenesis

The plasmid pBRT7Qβ was used to engineer two single-mutant viruses containing either mutation U830C (virus Qβ_U830C_) or C2011A (virus Qβ_C2011A_). Mutagenesis was carried out using the QuickChange Lightning Site-Directed Mutagenesis Kit (Agilent Technologies) with the primers 5′GACAATCTGTACCCTGCCGCTGCTTACTTTAAACTGAAA3′ (for Qβ_U830C_) and 5′TGGGATTCTCGGCTTAGTTATAACACGT TCCGCGG3′ (for Qβ_C2011A_), together with their respective complementary containing the mutations to introduce in the virus genome. The procedures to build and isolate the site-directed mutants were the same as previously described for other Qβ mutants (Arribas et al., 2018). A lytic plaque generated in *E. coli* Hfr by each of the mutants was picked and sequenced to test the presence of the desired mutation and the absence of any other that might have arisen during the mutagenesis process.

### 2.5. Plaque size assays

Areas of the lysis plaques formed by the viruses Qβ_Anc_ and the mutants Qβ_U830C_ and Qβ_C2011A_ were quantified in the following way. Around 100 pfu of each virus were mixed with ∼3 × 10^8^ bacteria and 3 ml of semisolid agar, and plated in agar plaques that had been prepared using 30 ml of the same preparation of LB-agar (Invitrogen). Plaques were incubated overnight at 37°C. Images of the Petri dishes were taken with ChemiDoc MP Imaging System (Bio-Rad) using a white sample tray and “Coomasie Blue” mode. Images were analyzed with Image Lab Software (Bio-Rad) (RRID:SCR_014210), using the tool “Volume Tool-Round” to manually select 70 lysis plaques of each virus whose areas were calculated. We used the Mann-Whitney test to assess the statistical significance of the differences between different sets of data.

### 2.6. Determination of virus entry into bacteria

Independent triplicate liquid 1 ml cultures, containing bacteria (3 × 10^8^ cfu/ml) and 10^5^ pfu of the indicated virus, were incubated at either 30, 37, or 43°C with low shaking (75 rpm) for 0, 5, 10, 15, and 20 min. At each time, 100 μL of EDTA 100 mM were added to stop virus entry. A control experiment showed that incubation of Qβ with this EDTA concentration completely inhibited its entry into *E. coli*, which agrees with previous results obtained for other phages (Paranchych, 1966). Cultures were centrifuged at 13000 × *g* at 4°C and the supernatant was discarded. The pellet was washed once with 500 μL of cold phage buffer containing EDTA 10 mM and re-suspended in phage buffer without EDTA. The number of infectious centers was estimated by plaque assay. Parallel cultures that were incubated at 4°C in the presence of EDTA 10 mM were used to estimate the virus background. The experimental points were fitted to a model of the form:

*P* = *P*_0_ – *P*_0_e^–^*^cNt^*

where *P* is the number of infectious centers, *P*_0_ is the number of pfu used in the experiment (10^5^ pfu), *N* is the number of bacteria in the sample (3 × 10^8^ cfu), and *t* is the incubation time. We defined the parameter *k* (expressed in ml/min/cell), corresponding to *c/N*, as the constant of formation of infectious centers at the bacteria density assayed. Fittings were performed using the function NonLinearFit of Mathematica 9.0 (Wolfram Research). Viruses whose *k*-values were compared were always assayed in the same experiment, using the same cell preparation. Differences were considered statistically significant when the value obtained for a particular virus was outside the confidence interval of the one to which it was compared.

Additional experiments were performed in the same way but with a fixed incubation time (10 min) and using different bacterial concentration (indicated in the corresponding figure). The statistical significance of the differences between the number of infectious centers obtained in different sets of data was assayed by means of the Student’s *t*-test.

### 2.7. One step growth curves

Duplicate 1 ml liquid cultures containing 3 × 10^8^ bacteria and 10^6^ pfu of the viruses Qβ_Anc_, Qβ_U830C_, or Qβ_C2011A_ were incubated for 5 min (curves performed at 30 and 37°C) or 10 min (curves performed at 43°C) in NB medium and 75 rpm. The reason for incubating longer at 43°C was to allow the entry of a greater amount of virus to mitigate the low replication yield at this temperature. In all cases, virus entry was stopped by a 10000-fold dilution of the cultures. Diluted cultures were incubated at the temperature assayed in a static bath, and only mixed by inversion right before taking the samples, to avoid additional encounters between cells and viruses. At different times, 250 μL aliquots were removed and centrifuged to obtain the virus supernatants that were titrated. The duration of the latent period was calculated as the intercept between the regression line of the natural logarithm of the virus titers vs. time during the exponential rise period and the regression line for the points corresponding to the pre-rise period.

### 2.8. Burst size determination

Triplicate 1 ml liquid cultures containing 3 × 10^8^ bacteria and 10^6^ pfu of the virus indicated were incubated for 10 min at either 30, 37, or 43°C in NB medium and 75 rpm to allow virus entry into bacteria. After this time, a 0.3 ml aliquot was removed to determine the number of infectious centers as described above (see section “2.6. Determination of virus entry into bacteria”). Another 0.3 ml were diluted 100000-fold (for the assays carried out at 30 and 37°C) or 10000-fold (for the assays carried out at 43°C, which rendered lower titers), and incubated at the same temperature used for virus entry with shaking (250 rpm). The incubation times were 75, 55, and 75 min for Qβ_Anc_ and Qβ_C2011A_ at 30, 37, and 43°C, respectively, and 90, 80, and 75 min for Qβ_U830C_ at 30, 37, and 43°C, respectively. The times were chosen so that each virus would have reached the plateau as shown in the one step growth curves. After these times, the virus supernatants were collected and titrated to estimate the amount of extracellular virus. The burst size was calculated by dividing the virus titers (multiplied by the initial dilution factor) by the number of infectious centers determined in the aliquot removed after the initial 10 min allowed for virus entry.

### 2.9. Preservation of Qβ infectivity in cellular lysates

To study the virus interaction with cellular debris, cellular lysates were prepared by transferring 10 ml of a stationary culture of *E. coli* Hfr into a 50 ml flask. The culture was treated with 500 μL of chloroform at 25°C, 250 rpm for 20 min. To completely break the cells, the flask was vortexed at maximum speed for 5 extra minutes. Finally, lysates were centrifuged at 25°C, 12000 rpm for 10 min. A 300 μL fraction of the supernatant was plated on an NB plate to check that there were no live bacteria remaining that could allow virus replication. Triplicates containing 1 ml of lysate supernatant were incubated with 3 × 10^5^ pfu for 16 h at 30, 37, or 43°C. Controls for each temperature were prepared the same way, but using NB instead of lysate supernatant. Test samples and controls were titrated by plaque assay.

### 2.10. RNA extraction, cDNA synthesis, PCR amplification, and nucleotide sequencing

Viral RNA was prepared following standard procedures to determine the consensus sequence either from biological clones or from complex virus populations. RNAs were used for cDNA synthesis with the avian myeloblastosis virus reverse transcriptase (Promega), followed by PCR amplification using Expand high-fidelity DNA polymerase (Roche). The pairs of oligonucleotide primers used for RT-PCR were the following: P1 forward (5′CTTTAGGGGGTCACCTCACAC3′) with P1 reverse (5′GGATGGGTCACAAGAACCGT3′) to amplify from nucleotide position 10 to 1595, P2 forward (5′GACGT GACATCCGGCTCAAA3′) with P2 reverse (5′CAACGGACGGAACATCTCCT3′) to amplify from nucleotide position 1109 to 2787 and P3 forward (5′GTGCCATACCGTTTGACT3′) with P3 reverse (5′GATCCCCCTCTCACTCGT3′) to amplify from nucleotide position 2254 to 4195. PCR products were column purified (Qiagen) and subjected to standard Sanger sequencing using Big Dye Chemistry with an automated sequencer (Applied Biosystems; Perkin Elmer). Sequences were assembled and aligned with Geneious Pro v4.8.5.^2^ Mutations relative to the sequence of the virus Qβ_Anc_ were identified using the same software.

### 2.11. Statistical analysis

All statistical analyses were performed with the program Mathematica 9.0 (Wolfram Research). When calculating the statistical significance of the differences between two series of data, the Location-Test function was used to choose the most appropriate test for the comparison, which in all cases, except for the comparison of the size of the lysis plaques produced by different viruses, was the *t*-test (Function *T*-Test).

## 3. Results

### 3.1. Adaptation of bacteriophage Qβ to low host density and suboptimal temperatures

In our previous publication (Laguna-Castro and Lázaro, 2022), we found that the optimal bacterial density for the growth of bacteriophage Qβ at 37°C under the standard conditions used in our laboratory (2 h incubation with bacteria grown to an optical density at 600 nm of 0.8) was 3 × 10^8^ cfu/ml. To analyze the effect of temperature on phage replication as a function of the host density, the phage was propagated for 16 transfers at either 30 or 43°C. For each temperature, we tested the following concentrations of bacteria: 3 × 10^8^, 3 × 10^7^, 3 × 10^6^, and 3 × 10^5^ cfu/ml, performing two replicas for each (Figure 1). Since at 37°C the virus could not be maintained when bacterial density was 3 × 10^4^ cfu/ml, in this new experiment we omitted that condition. In this way, we obtained 8 evolutionary lineages for each temperature that were designated with a nomenclature indicating the bacteria concentration used in the transfers, the replication temperature and the replica number (see section “2.2. Evolution experiment” and Figure 1). The growth curve of *E. coli* (not infected with Qβ) as a function of temperature is shown in Supplementary Figure 1. It can be seen that, while growth at 37 and 43°C is quite similar, at 30°C it is much slower.

**FIGURE 2:**
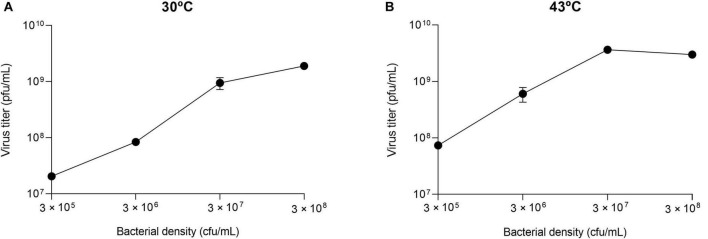
Virus titers as a function of the bacterial density. The values represented correspond to the first transfer performed in the evolution experiment (Figure 1). They were obtained by infecting independent cultures containing the bacterial densities indicated with 10^7^ pfu of the virus Qβ_Anc_. Error bars represent the standard deviation of the two replicas of the experiment. **(A)** Replication temperature of 30°C. **(B)** Replication temperature of 43°C.

The results obtained in the first transfer, which in all cases was initiated from the same virus sample (virus Qβ_Anc_), were used to determine whether the optimal bacterial density for Qβ replication depended on temperature. At 30°C, we observed that the curve flattened between 3 × 10^7^ and 3 × 10^8^ cfu/ml, and at 43°C the maximum virus titer was obtained at 3 × 10^7^ cfu/ml (Figure 2). It should be noted that, since the optimal bacterial density varies with the number of viruses (Laguna-Castro and Lázaro, 2022), these results are only valid when 10^7^ pfu are used in the experiment. The virus was propagated for 16 transfers at all but the lowest bacterial density (3 × 10^5^ cfu/ml). In that case, the virus was extinguished at transfers 9 and 10 for the replicas performed at 30°C, and at transfer 7 for those performed at 43°C. As it happened during the propagation of Qβ at 37°C at lower-than-optimal bacterial densities, the evolutionary lineages (with the only exception of the two replicas propagated at 43°C at 3 × 10^8^ cfu/ml) became progressively enriched in viruses that gave rise to lysis plaques with smaller-than-usual size. In the following sections we will analyze this character quantitatively.

**FIGURE 3:**
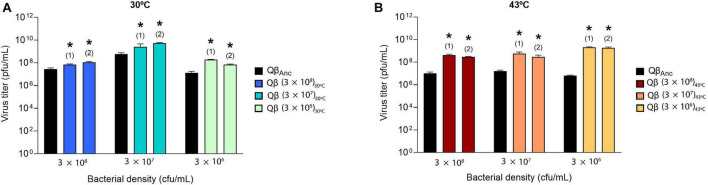
Replicative ability of the lineages obtained at transfer number 16 in the evolution experiment (Figure 1). The assay was performed as described in section “2.3. Determination of the virus replicative ability”. Each evolutionary lineage was assayed at the same bacterial density at which it had evolved (indicated at the bottom of the figure). Each bar represents the average of three replicas. The color code used to distinguish the different lineages is the same shown in Figure 1. The virus Qβ_Anc_, which was assayed at all host densities, is shown in black. **(A)** Replication temperature of 30°C. **(B)** Replication temperature of 43°C. Asterisks above the bars indicate that the result is significantly different from the value obtained for the virus Qβ_Anc_ (*p* < 0.05) according to a Student’s *t*-test. The raw data of the experiment can be found in Supplementary Tables 1, 2.

We then determined whether the populations obtained at transfer 16 had increased their replicative ability with respect to the ancestor. To ensure that viral production was not saturated during the assay, this was performed with 10^4^ pfu instead of 10^7^ pfu (the amount used in the transfers). The results obtained (Figure 3) showed that all evolved populations rendered significantly higher titers than the ancestor (*p* < 0.05 for all comparisons between the ancestor and the evolved populations, *t*-test).

### 3.2. Determination of the consensus sequence of the adapted populations

**FIGURE 4:**
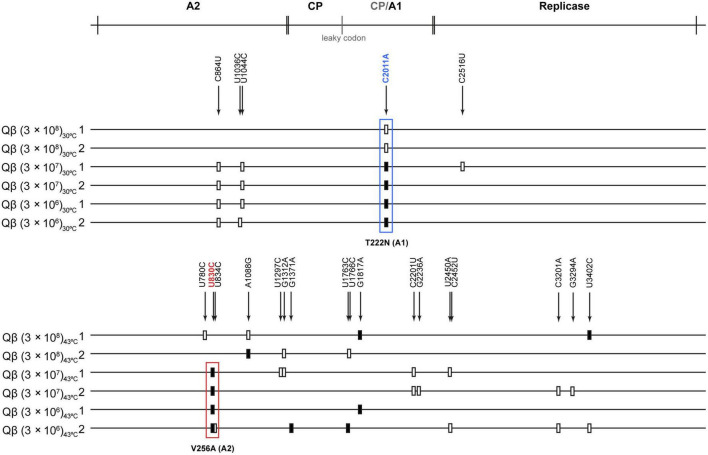
Mutations detected in the consensus sequence of the evolutionary lineages at transfer 16. The scheme above the figure represents the Qβ genome with the proteins encoded in it (CP means capsid protein and CP/A1 the additional extension present in the A1 protein). Mutations relative to the sequence of the virus Qβ_Anc_ are marked with a filled rectangle (fixed mutations) or with a blank rectangle (polymorphic mutations). The exact position of each mutation is indicated by numbers above the lines representing the genomes of populations evolved either at 30 or 43°C (indicated on the left of each line). The most frequently detected mutation during evolution at 30°C, (C2011A, T222N in the A1 protein) is marked with a blue rectangle, whereas the most common mutation during evolution at 43°C (U830C, V256A in the A2 protein) is marked with a red rectangle.

To determine the genetic changes responsible for adaptation to the assayed conditions, we determined the consensus sequences of all evolutionary lineages obtained at transfer 16 (Figure 4). The most remarkable result obtained at 30°C was that all lineages selected the mutation C2011A (T222N in the A1 protein). This mutation was also selected in our previous experiment of evolution at 37°C, although in that case it only appeared in the lineages evolved at lower-than-optimal bacterial density. Other mutations that appeared as polymorphisms at 30°C and lower-than-optimal bacterial densities were C864U and U1044C (both synonymous), which were also detectable in some of the lineages evolved at 37°C (Laguna-Castro and Lázaro, 2022).

The results obtained at 43°C showed interesting differences with those observed at 30 and 37°C. First, mutation C2011A was not selected in any lineage. In contrast, in all those propagated at bacterial densities below 3 × 10^8^ cfu/ml, mutation U830C (V256A in the A2 protein) was present. In addition, other mutations, some of which had previously been identified in other experiments of Qβ adaptation to high temperature and standard bacterial density, were also detected (Arribas et al., 2014; Somovilla et al., 2019, 2022; Arribas and Lázaro, 2021). The presence of these mutations shows that the phage is able to adapt to increased temperature as long as the bacterial density is above 3 × 10^5^ cfu/ml. It is interesting that mutation A1088G (D342G in the A2 protein), which was always selected in our lab when Qβ was adapted to 43°C using bacterial densities around 3 × 10^8^ cfu/ml, was not selected when the amount of available bacteria was lower.

### 3.3. Selective value of mutations U830C and C2011A

**FIGURE 5:**
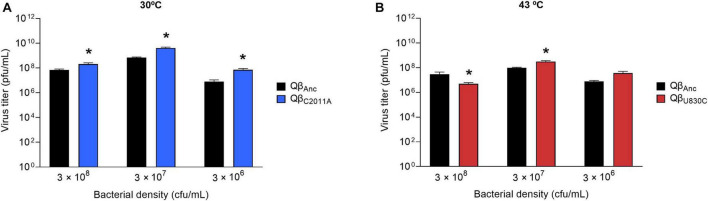
Replicative ability of the mutants Qβ_U830C_ and Qβ_C2011A_ under selective conditions. The assay was performed as described in section “2.3. Determination of the virus replicative ability”. **(A)** Mutant Qβ_C2011A_ (blue bars) replicating at 30°C and different bacterial densities. **(B)** Mutant Qβ_U830C_ (red bars) replicating at 43°C and different bacterial densities. In both cases, the virus Qβ_Anc_ is shown with black bars. Each bar represents the average of three replicas. Asterisks above the bars indicate that the result obtained for the mutant is significantly different from that obtained for the virus Qβ_Anc_ (*p* < 0.05) according to a Student’s *t*-test. The raw data of the experiment can be found in Supplementary Tables 3, 4.

**FIGURE 6:**
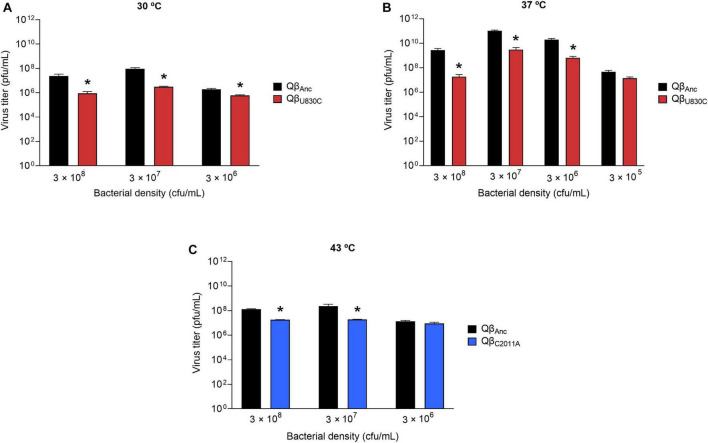
Replicative ability of the mutants Qβ_C2011A_ and Qβ_U830C_ under restrictive conditions. The assay was performed as described in section “2.3. Determination of the virus replicative ability”. **(A)** Mutant Qβ_U830C_ (red bars) replicating at 30°C. **(B)** Mutant Qβ_U830C_ (red bars) replicating at 37°C. **(C)** Mutant Qβ_C2011A_ (blue bars) replicating at 43°C. In all cases, replication took place at different bacterial densities. The virus Qβ_Anc_ is always shown with black bars. Each bar represents the average of three replicas. Asterisks above the bars indicate that the result obtained for the mutant is significantly different from that obtained for the virus Qβ_Anc_ (*p* < 0.05) according to a Student’s *t*-test. The raw data of the experiment can be found in Supplementary Tables 5–7.

For a more detailed analysis of the adaptive advantages of mutations U830C and C2011A, we used the Qβ expression vector pBRT7Qβ and specific primers to obtain mutant viruses that only contained mutation U830C (virus Qβ_U830C_) or C2011A (virus Qβ_C2011A_). We determined the virus titers obtained in a replication assay carried out with the single mutants at both selective and non-selective temperatures at all the bacteria concentrations compatible with the preservation of the virus population for the 16 transfers of the evolution experiment (Figures 5, 6).

The virus yields obtained at selective temperatures (43°C for Qβ_U830C_ and 30°C for Qβ_C2011A_) showed that mutation U830C only increased significantly the virus titers at 43°C at 3 × 10^7^ cfu/ml (Figure 5). At other bacterial densities the mutant showed significantly lower titers than the ancestor (3 × 10^8^ cfu/ml) or the difference was non-significant (3 × 10^6^ cfu/ml), suggesting that the presence of this single mutation is not sufficient for the virus to adapt to both selective pressures, high temperature and low host density, simultaneously. Mutation C2011A increased the replicative ability with respect to the virus lacking the mutation at all bacteria densities assayed at 30°C.

In contrast to the results above, at non-selective temperatures (30 and 37°C for Qβ_U830C_; 43°C for Qβ_C2011A_), both mutations always rendered lower virus yields than the ancestor (Figure 6), suggesting that they probably have a fitness cost under those conditions. Another observation was that the lower the bacterial density assayed the lower the differences between the replicative ability of the ancestor and each of the mutants. In fact, at 43°C and 3 × 10^6^ cfu/ml the differences between the mutant Qβ_C2011A_ and the ancestor were non-significant. The same happened with the mutant Qβ_U830C_ when it was compared with the ancestor at a bacterial density of 3 × 10^5^ cfu/ml at 37°C. We included this condition because it was also compatible with the propagation of the virus Qβ_Anc_.

As it happened in the case of mutation C2011A, to confirm whether mutation U830C was also responsible for the smaller size of the plaques observed in most of the evolved lineages at 43°C, we determined the area of 70 lytic plaques produced by each of the viruses Qβ_U830C_ and Qβ_Anc_, using the Image Lab Software. The average size of the plaques produced by the ancestor was 3.04 ± 1.31 mm^2^, which was significantly higher than that of the plaques produced by Qβ_U830C_ (0.15 ± 0.09 mm^2^) (*p* = 3.1 × 10^–25^, Mann-Whitney test). It is remarkable that in all assays carried out to determine the replicative ability of the mutant Qβ_U830C_ (Figures 5, 6) we always obtained a certain amount of normal-size plaques, which increased with the bacterial density. The only exception was the assay performed at 43°C and 3 × 10^6^ cfu/ml that always rendered small plaques.

In order to find out the reasons of the selective advantages of mutations U830C and C2011A, as well as their possible fitness costs under the conditions that did not promote their selection, we determined several of the parameters that define the viral infection cycle. The results obtained are detailed in the following section.

### 3.4. Effect of mutations U830C and C2011A on the parameters defining the Qβ infection cycle

To check the effect of mutations U830C and C2011A on the ability of Qβ to infect *E. coli*, we first performed an assay in which we determined the values of the constant of formation of infectious centers (*k*; see section “2.6. Determination of virus entry into bacteria”) for the virus Qβ_Anc_ at 30, 37, and 43°C. We observed that this virus generated infectious centers more rapidly at 43°C than at 37°C, and the lowest value was obtained at 30°C (see the three first rows in Table 1). The results are in agreement with a greater capacity for encounters between viruses and bacteria at high temperatures. Note that *k* only indicates how rapidly bacteria are infected without distinguishing between efficiency of adsorption to the pili and efficiency of internalization of the phage genomes.

**TABLE 1:** Values of the constant of formation of infectious centers (*k*) for the viruses Qβ_Anc_, Qβ_U830C_, and Qβ_C2011A_ at different temperatures.

Virus^1^	Temperature	*k* (ml/min/cell)^2^	*p*-value^3^	*R* ^2^
Qβ_Anc_*^4^	30°C	3.7 × 10^–11^	0.002	0.99
Qβ_Anc_*	37°C	1,4 × 10^–10^	0.002	0.98
Qβ_Anc_*	43°C	3.7 × 10^–10^	<0.001	0.99

Qβ_Anc_	30°C	2.7 × 10^–11^	0.001	0.99
Qβ_U830C_*	30°C	2.0 × 10^–10^	<0.001	0.99

Qβ_Anc_	37°C	1.2 × 10^–10^	0.001	0.99
Qβ_U830C_*	37°C	2 × 10^–10^	<0.001	0.99

Qβ_Anc_	43°C	4.3 × 10^–10^	0.001	0.97
Qβ_U830C_*	43°C	8.3 × 10^–10^	0.001	0.99

Qβ_Anc_	30°C	6.3 × 10^–11^	<0.001	0.99
Qβ_C2011A_*	30°C	5.7 × 10^–10^	0.002	0.98

Qβ_Anc_	37°C	1.3 × 10^–10^	0.015	0.96
Qβ_C2011A_	37°C	3.3 × 10^–10^	0.014	0.93

Qβ_Anc_	43°C	3.2 × 10^–10^	0.002	0.97
Qβ_C2011A_*	43°C	5.3 × 10^–10^	<0.001	0.98

^1^Each group of viruses separated by a gray row were tested in the same assay.

^2^*k* is defined in section “2.6. Determination of virus entry into bacteria”.

^3^The *p* and *R*^2^ values indicate the statistical significance of the fitting of the experimental points to the function indicated in section “2.6. Determination of virus entry into bacteria”.

^4^The asterisk indicates whether differences between the k-values for the viruses included in the same group were statistically significant.

The criterion followed was that the k-value for a particular virus was outside the confidence interval of the others to which it was compared.

Then, we compared the value of the constants obtained for the virus Qβ_Anc_ with those corresponding to the viruses Qβ_U830C_ and Qβ_C2011A_ at 30, 37, and 43°C (Table 1). Each comparison between a given mutant and the ancestor was performed in the same experiment, to avoid differences due to the state of the bacteria on different days. Differences between constants were considered significant when their confidence intervals did not overlap. The main conclusion was that both mutants increased the velocity of formation of infectious centers at the three temperatures tested. The greatest effect for both mutations was observed at 30°C, which is the temperature at which the virus infected the bacteria worst. The only difference that was non-significant (Qβ_Anc_ and Qβ_C2011A_ at 37°C) was probably due to the poorer fit of the experimental points to the function used, resulting in wider confidence intervals.

We also performed a complementary assay in which the viruses Qβ_Anc_, Qβ_U830C_, and Qβ_C2011A_ were incubated for a fixed time (10 min) with different bacterial densities, after which the number of infectious centers formed was determined. The results obtained (Supplementary Figure 2) showed that both mutations significantly increased the formation of infectious centers at all cell densities assayed. Altogether, the results suggest that at least a part of the beneficial effect of the mutations U830C and C2011A might be due to a greater entry of Qβ into the bacteria.

**FIGURE 7:**
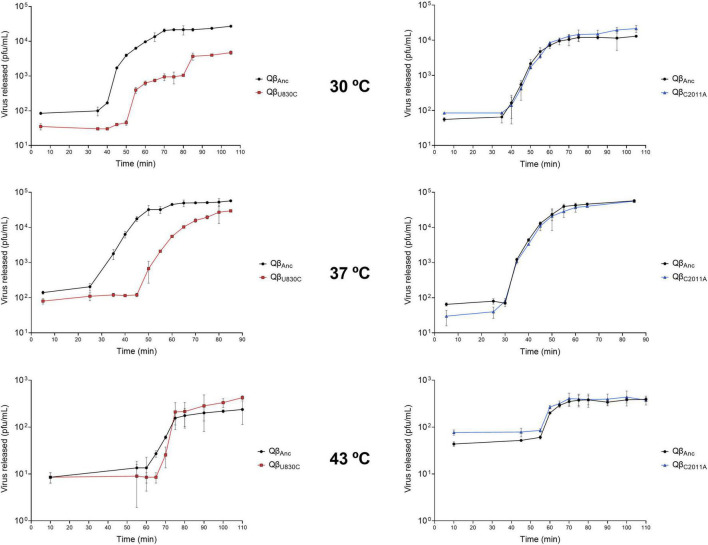
One step growth curves obtained for the viruses Qβ_Anc_, Qβ_U830C_ and Qβ_C2011A_ replicating at 30, 37, and 43°C. The details of the experiment are described in section “2.7. One step growth curves”.

Next, we performed one step growth curves to compare the latent period of the ancestor with those of the viruses Qβ_U830C_ and Qβ_C2011A_ at 30, 37, and 43°C (Figure 7). Curves represented in the same graphic were performed in the same assay, using the same preparation of bacteria. Since these experiments involve the handling of a large number of samples separated by a short period of time, they are subject to some error which is probably the cause of the small differences in the estimated latent period for the ancestor in different tests. Despite these difficulties, there are some clear results. First, the shortest latent period for the ancestor was obtained at 37°C (between 25 and 30 min). At 30°C it increased to ∼ 35 min, and at 43°C it becomes considerably longer (about 60 min). While mutation C2011A did not produce any appreciable change in the duration of the latent period at any of the temperatures assayed, the same was not true for U830C, which greatly increased it at all temperatures, with the smallest effect being at 43°C. The large increase in the time required for the release of the phage progeny is possibly the reason why this mutation is not selected neither at 30°C nor at 37°C. The lower effect it has at 43°C is probably what makes it an acceptable option at this temperature. It is not clear why mutation C2011A, which does not increase the latent period at 43°C, is not selected at this temperature, especially if it is taken into account that it increases the adsorption rate similarly to U830C.

A remarkable observation is that, at 30°C, the release of progeny produced by the mutant Qβ_U830C_ took place in two phases. It is intriguing that in the first phase all the lysis plaques were of normal size, while in the second phase all of them had smaller-than-usual size. At 37°C, the same virus produced a mixture of lysis plaques of both sizes, while at 43°C, all plaques were small.

Finally, we determined the burst size produced by the ancestor and the mutant viruses Qβ_U830C_ and Qβ_C2011A_ at 30, 37, and 43°C (Table 2). The results show that both mutations produced significant decreases in this parameter, with the only exception of U830C at 43°C that caused a small increase. Given the low viral production at this temperature, it is hard to say if it has any biological significance.

**TABLE 2:** Burst size of viruses Qβ_Anc_, Qβ_U830C_, and Qβ_C2011A_ at different temperatures.

Virus^1^	Temperature	Burst size (pfu)
Qβ_Anc_	30°C	3530 ± 586
Qβ_U830C_*^2^	30°C	603 ± 335

Qβ_Anc_	37°C	761 ± 42
Qβ_U830C_*	37°C	591 ± 70

Qβ_Anc_	43°C	2.1 ± 0.1
Qβ_U830C_*	43°C	6.5 ± 0.6

Qβ_Anc_	30°C	3180 ± 273
Qβ_C2011A_*	30°C	948 ± 152

Qβ_Anc_	37°C	764 ± 75
Qβ_C2011A_*	37°C	411 ± 27

Qβ_Anc_	43°C	2.1 ± 0.1
Qβ_C2011A_	43°C	2.3 ± 0.5

^1^Each group of viruses separated by a gray row were tested in the same assay.

^2^The asterisk indicates that the difference in the burst size of the two viruses included in the same group is statistically significant (*p* < 0.05, Student’s *t*-test).

### 3.5. Preservation of virus infectivity in different environments

Another feature influencing virus fitness is the time that infectivity can be preserved in the extracellular environment in the period between infections. Previous assays carried out with the virus Qβ_Anc_ showed that infectivity did not decrease after 2 h of incubation in NB medium in the absence of bacteria at either 37 or 43°C. However, other observations carried out in our lab showed that prolonged incubation of Qβ with bacteria in a replication assay carried out at 43°C resulted in an initial increase in the virus titers (which lasted for about 5–6 h) followed by a sharp decline until almost the total elimination of viral infectivity at 24 h. In contrast to this, different amounts of virus incubated for 24 h in NB medium at 43°C did not show any significant decrease in their titers. The result suggests that Qβ loses infectivity faster when it is replicating in a medium with bacteria than in cell-free NB medium.

**FIGURE 8:**
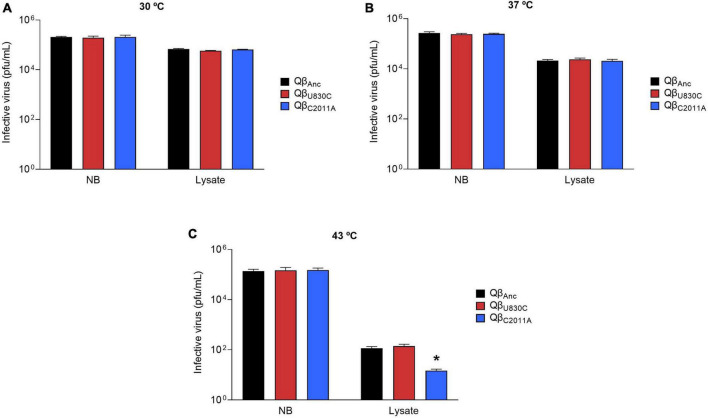
Infectivity of viruses Qβ_Anc_, Qβ_U830C_, and Qβ_C2011A_ after incubation in NB medium or a cell lysate in the absence of live bacteria. The assay was performed as described in section “2.9. Preservation of Qβ infectivity in cellular lysates”. The virus Qβ_Anc_ is represented with black bars, Qβ_U830C_ with red bars and Qβ_C2011A_ with blue bars. Each bar represents the average of three replicas. **(A)** Incubation temperature of 30°C. **(B)** Incubation temperature of 37°C. **(C)** Incubation temperature of 43°C. The asterisk above the bar corresponding to the virus Qβ_C2011A_ indicates that the result obtained is significantly different from those obtained for the viruses Qβ_Anc_ and Qβ_U830C_ (*p* < 0.05) according to a Student’s *t*-test. The raw data of the experiment can be found in Supplementary Table 8.

The experiment above described can be confounding because, at the same time that a fraction of the virus is losing its infectivity, another fraction is reproducing, and it is not possible to disentangle both processes. To prevent viral replication, we prepared a bacterial lysate artificially (see section “2.9. Preservation of Qβ infectivity in cellular lysates”), and incubated it with about 3 × 10^5^pfu of the ancestral virus or the mutants Qβ_U830C_ and Qβ_C2011A_ for 16 h at 30, 37, and 43°C, after which the virus infectivity in each sample was assayed. The results (Figure 8) showed that whereas all viruses kept most of their infectivity when incubated in NB medium, all they lost a considerable part of it when incubated with the cell lysate. The decay was temperature dependent, showing the largest effect at 43°C. The most interesting result was that the decrease at 43°C was significantly higher for the mutant Qβ_C2011A_ than for the ancestor or the mutant Qβ_U830C_. These findings could provide an explanation, which will be exposed in detail in the Discussion, to the question of why mutation C2011A is not selected at 43°C.

### 3.6. New evolution experiments from viruses Qβ_U830C_ and Qβ_C2011A_

**FIGURE 9:**
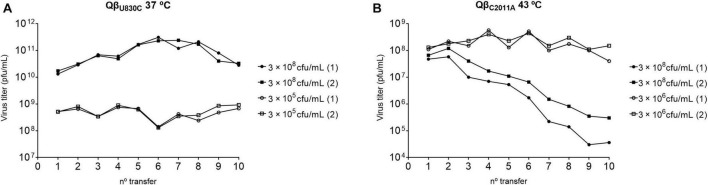
Virus titers obtained during the evolution of viruses Qβ_U830C_ and Qβ_C2011A_. **(A)** Mutant Qβ_U830C_ propagated at 37°C at the bacterial densities indicated in the margin of the figure. **(B)** Mutant Qβ_C2011A_ propagated at 43°C at the bacterial densities indicated in the margin of the figure. In all cases the experiment was initiated with 10^7^ pfu. After 2 h of incubation under the conditions indicated, the virus supernatants were collected, and a fraction of the phage suspension (10^7^ pfu or, when the titers did not allow it, the amount of phage contained in 100 μL of the previous supernatant) was used to initiate the following transfer and so on. There are two replicas for each condition (indicated with the numbers 1 and 2).

To better understand the fitness advantages and costs that determine the preferential selection of particular mutations depending on temperature, we performed two new evolution experiments that were carried out in duplicate. One of them consisted in the propagation of the mutant Qβ_U830C_ at 37°C (Figure 9A), a temperature at which it causes significant decreases in the growth rate (see Figure 6). The bacterial density was high (3 × 10^8^ cfu/ml) or low (3 × 10^5^ cfu/ml). In both conditions, the virus experienced an initial increase in its titers, which was greater at high bacterial density, and remained fairly constant during the rest of transfers. The consensus sequences showed the loss of mutation U830C in the two replicas carried out at high bacterial density and the permanence of the mutation when the bacterial density was low (Table 3). Surprisingly, mutation C2011A did not appear under the latter condition. The results are consistent with the non-significant fitness cost of U830C at 37°C when the bacterial density was 3 × 10^5^ cfu/ml (Figure 6). The initial presence of this mutation seems to confer a sufficient advantage that makes selection of C2011A unnecessary.

**TABLE 3:** Mutations present in the consensus sequence of the lineages propagated from viruses Qβ_U830C_ and Qβ_C2011A_ for 10 transfers under the conditions indicated.

Virus^1^	Bacterial concentration	Temperature	Mutations^2^
Qβ_U830C_ (1)	3 × 10^8^	37°C	–
Qβ_U830C_ (2)	3 × 10^8^	37°C	–
Qβ_U830C_ (1)	3 × 10^5^	37°C	U830C
Qβ_U830C_ (2)	3 × 10^5^	37°C	U830C
Qβ_C2011A_ (1)	3 × 10^8^	43°C	G1820A
Qβ_C2011A_ (2)	3 × 10^8^	43°C	–
Qβ_C2011A_ (1)	3 × 10^6^	43°C	U830C C2011A
Qβ_C2011A_ (2)	3 × 10^6^	43°C	U830C C2011A

^1^The number (1) and (2) indicate the two replicas performed for each evolutionary lineage.

^2^The sign—means that there is no mutations relative to the sequence of the wild-type virus.

The other evolution experiment was initiated with the mutant Qβ_C2011A_, which was propagated for 10 transfers at 43°C (a temperature at which this mutation is not selected), using a high (3 × 10^8^ cfu/ml) or a low (3 × 10^6^ cfu/ml) bacterial density (Figure 9B). The results showed that, at high bacterial density, the titers decreased until they reached values between 10^4^ and 10^6^ pfu/ml. However, propagation of the same virus at low bacterial densities produced an early increase in titers (up to approximately 10^8^ pfu/ml) that remained constant with some fluctuations throughout the experiment. Determination of the consensus sequences at transfer number 10 showed that, at high bacterial density, mutation C2011A was lost in both replicas, whereas at low bacteria density, the mutation could be preserved, although it was always accompanied by U830C (see Table 3). Therefore, it seems that mutation C2011A can be maintained at 43°C, as long as the bacterial density is low and mutation U830C is selected. The permanence of mutation C2011A agrees with the non-significant fitness cost of this mutation at 43°C when the bacterial density is 3 × 10^6^ cfu/ml (Figure 6).

## 4. Discussion

In a previous work, we showed that bacteriophage Qβ was able to improve its replicative capacity in the presence of lower bacterial concentrations than the one usually used in our laboratory at the optimal temperature for virus replication (37°C) (Laguna-Castro and Lázaro, 2022). In all cases, adaptation occurred through a mutation in the A1 protein that favored entry of the virus into the bacteria, while at the same time there seemed to be a decrease in burst size. This lower viral production per infected bacterium could be the reason why, when the concentration of bacteria was optimal, the mutation was not selected.

Since both virus entry into bacteria and replication to generate a progeny can be strongly affected by temperature, in this work we wanted to study the adaptive strategies employed by Qβ when the concentration of bacteria was decreased at two temperatures different from the optimum: one lower (30°C) and the other one higher (43°C). It is noteworthy that, in both cases, the curves of viral yield as a function of bacterial density approached a plateau at about 3 × 10^7^ cfu/ml. From this point on, the increases were low (30°C) or turned into declines (43°C) (Figure 2). In this regard, it is necessary to point out that, since the incubation time at each transfer allows more than one infection round, it is difficult to give a precise value for the optimal bacterial density. Depending on the duration of the infection cycle at each temperature, after 2 h there could be a different amount of viruses that have produced a progeny in new infection rounds or are in the eclipse phase, thus being undetectable in a plaque assay.

The first remarkable result we obtained in the evolution experiment was that, at both temperatures, Qβ was able to increase its replicative capacity at all bacterial densities assayed but 3 × 10^5^ cfu/ml. Under this condition, the virus was extinguished in both replicas carried out either at 30 or 43°C, probably due to the low viral production when two selective pressures (suboptimal temperature and low host availability) are operating together. As it happened in the experiment carried out at 37°C, another observable phenotypic feature was that in all lineages evolved at 30 and 43°C [with the only exception of the two replicas of Qβ (3 × 10^8^)_43°C_] as the number of transfers increased the lysis plaques became smaller than usual. There are several theoretical and experimental studies that attempt to relate the changes in the parameters defining the viral infective cycle with the size of the lysis plaques (Abedon and Culler, 2007; Shao and Wang, 2008; Abedon and Yin, 2009; Gallet et al., 2011). Although the results are not easy to interpret and do not always coincide, in some cases the increases in the adsorption rate were related to decreases in plaque size (Shao and Wang, 2008; Gallet et al., 2011).

Sequencing of the evolved populations showed another interesting result, which was that all lineages evolved at 30°C (including those propagated at the highest bacterial density: 3 × 10^8^ cfu/ml) selected mutation C2011A in the A1 protein, similarly to Qβ evolved at 37°C and bacterial densities lower than 3 × 10^8^ cfu/ml (Laguna-Castro and Lázaro, 2022). In contrast to this, all lineages evolved at 43°C and bacterial densities below 3 × 10^8^ cfu/ml did not select mutation C2011A and, instead of it, presented another mutation, U830C, located in the A2 protein. This protein, which is present in a single copy (Gorzelnik et al., 2016), is considered to be the virus component that interacts with the bacterial receptor (Manchak et al., 2002; Toropova et al., 2011; Rumnieks and Tars, 2017), allowing penetration of the virus genome into the cell, as it happens in other levivirus (Kozak and Nathans, 1971; Paranchych et al., 1971; Krahn et al., 1972; Shiba and Miyake, 1975; Reynolds and Paranchych, 1976). All evolutionary lineages presented additional mutations that might represent specific adaptations to the particular temperature assayed, independently of the bacterial density. The fact that most mutations found at 43°C had previously been described in adaptation experiments carried out with Qβ at this temperature agrees with this possibility.

Experiments carried out with single mutant viruses containing either U830C or C2011A showed that both mutations increased the formation of infectious centers at 30, 37, and 43°C, suggesting that the same adaptive mechanism is operating at all temperatures assayed (see Table 1 and Supplementary Figure 2). These findings raise two relevant questions. The first one is: Why is mutation C2011A selected at 30°C at a bacterial density at which it has a fitness cost at 37°C? The second question is: Why are mutations U830C and C2011A selected at different temperatures in an exclusive way?

To answer the first question, we determined the constant of formation of infectious centers for the virus Qβ_Anc_ at the three temperatures tested, finding that the lowest value was at 30°C, four times lower than at 37°C and ten times lower than at 43°C (Table 1). This result suggests that if the constant is low, as it happens at 30°C even when the bacterial density is high, the adaptive strategy employed by the virus is the same as that used when host availability is limited. The slower growth of *E. coli* at 30°C than at 37°C (Supplementary Figure 1) may also cause uninfected bacteria to reach lower densities at each transfer, so that the improvement of the interaction with the pilus through mutation C2011A may be an advantage. To investigate the second question, we estimated the replicative ability of the single mutant viruses, which allowed us to verify that mutation U830C had strong fitness costs at 30 and 37°C, while mutation C2011A had similar ones at 43°C (Figure 6). Determination of the latent period for both viruses suggested that the fitness cost at 30 and 37°C of mutation U830C was due to a strong increase in its duration at those temperatures (Figure 7). At 30°C, the release of the virus Qβ_U830C_ was biphasic, with a first phase in which all viruses produced normal-size plaques, and a second phase in which all plaques were smaller than usual. Since mutation U830C seemed to be the responsible of the generation of small-size plaques, this result, together with the heterogeneity in the size of the plaques observed in the replicative ability assays performed with the mutant Qβ_U830C_ (Figures 5, 6), suggests that this mutation can be selected against even in a single replication cycle. In that case, those viruses that lost the mutation earliest would be the first to exit the cell, giving rise to the first phase of the release period at 30°C. At 37°C, however, there was a single period of viral release, in which viruses produced a mixture of small and normal-size plaques. This may be due to the fact that, as the latent period at 37°C is shorter, there is no time for a separation into two phases, similar to that observed at 30°C. At 43°C, all viruses released produced small plaques, indicating that the fitness cost of mutation U830C at this temperature and high host density is not sufficient for its selection against in a single infection cycle.

What can be the reason for the increase in the latent period produced by mutation U830C? Its location in the A2 protein, which is multifunctional, offers interesting alternatives. In addition to interacting with the pili, A2 also binds to a cellular protein, MurA, which is involved in the synthesis of peptidoglycan by the bacterium (Brown et al., 1995). When sufficient A2 has been synthesized, its binding to MurA inhibits cell wall synthesis, opening holes, and eventually causing bacterial lysis (Karnik and Billeter, 1983; Winter and Gold, 1983; Bernhardt et al., 2001; Reed et al., 2012, 2013; Cui et al., 2017). It is possible that the increased latent period produced by mutation U830C is due to a worsening of the interaction of A2 with MurA at 30 and 37°C when the amino acid at position 256 is alanine, instead of valine. At 43°C, the temperature at which the latent period is longer, both amino acids would be almost equally “bad” for the interaction between A2 and MurA, giving rise to only small differences in the latent period. In support of this idea, it has been reported that incubation of Qβ with MurA inhibits infection, suggesting that the pilus and MurA bind to the same region of A2. Thus, improving interaction with the pilus could be at the cost of worsening the interaction with MurA (Reed et al., 2013). Comparison of the complexes generated between wild and mutant A2 with MurA at the three temperatures assayed in this study could explain the molecular mechanisms leading to our results. To this aim, molecular dynamics simulations, combined with microscopy techniques could be of great help (Jana and May, 2020; Bruinsma et al., 2021). In this sense, it is worth mentioning the works carried out with MS2 that have allowed to model not only the viral capsid, but also the gRNA, getting the first complete all-atom model of the virus (Farafonov and Nerukh, 2019; Farafonov et al., 2022).

It is more difficult to interpret why mutation C2011A, which also favors the formation of infectious centers at 43°C without prolonging the latent period, is not selected at this temperature. Our proposal for the mechanism of action of this mutation, located in the A1 protein, is that it provides primary binding sites to the pili (Laguna-Castro and Lázaro, 2022). According to some models, mostly inspired in studies carried out with the related phage MS2 that also uses the bacterial pili as receptor (Toropova et al., 2011; Dent et al., 2013), subsequent retraction of the pili would bring the attached viruses closer to the cell surface, thus facilitating the entry into the cell of the complex formed by Mat (the protein that interacts with the pilus in MS2) and the virus RNA (Meng et al., 2019; Harb et al., 2020). If a similar mechanism is operating in Qβ, it might happen that the empty viral capsids produced after genome internalization would remain in the extracellular environment, and the possibility that they could bind to new susceptible bacteria through the A1 protein cannot be ruled out. Additional studies, carried out with MS2 and Qβ, show that the pili that have undergone the retraction process are detached from the bacteria preventing further infections (Harb et al., 2020). It is quite probable that these free pili are able to bind viruses that otherwise could infect susceptible bacteria, as it has been demonstrated for phages R17 and M12 (Valentine and Strand, 1965; Paranchych et al., 1971). In both situations, the increase in the adsorption rate produced by mutation C2011A would be a disadvantage for the progression of the infection, as it could result in the “sequestration” of susceptible bacteria by empty capsids and of infective viruses by non-functional pili. Both processes would acquire greater relevance at high bacterial density, leading to more negative effects of the mutation. Since mutation U830C is located in the A2 protein, which is introduced into the cell together with the virus genome, it cannot have any effect in the adsorption of empty capsids to bacteria. The presence of A2 in a single copy probably also limits the process of virus binding to detached pili or other bacteria components. Degradation or binding of viruses by some of the bacterial components released after lysis might also contribute to reduce viral infectivity (Rabinovitch et al., 2003; Aviram and Rabinovitch, 2008).

Several assays in which Qβ_Anc_, Qβ_U830C_, and Qβ_C2011A_ were incubated with an artificial cell lysate at different temperatures for 16 h showed that the virus titers experienced a decline, which was of greater magnitude at 43°C than at 30 or 37°C (Figure 8), perhaps due to the highest value for the constant of formation of infectious centers at that temperature. It was also observed in similar assays that at 43°C (and only at this temperature), the virus containing mutation C2011A reduced its titers much more than the wild-type virus or the mutant containing U830C, which could be due to the mechanisms proposed above, providing in this way an explanation for the fitness costs of C2011A at 43°C. It could be argued that incubation with the lysate lasted longer than the time allowed for virus replication during the transfers in the evolution experiment. In addition, bacteria were lysed in a different way than that occurring during viral infection. We recognize these limitations, which could not be addressed in this study due to the difficulties to separate bacteria that have been lysed by the virus from those that have not been infected or have not completed the lysis process. Nevertheless, the aforementioned detachment of the pili, which might occur at a greater extension during Qβ infections, could increase the non-productive interactions with the virus. This fact would contribute to increase the negative effect of mutation C2011A at 43°C, which could thus be evident in a shorter time. We plan to develop a system to purify bacterial pili that allow us to compare their interaction with Qβ wild type and each of the mutants Qβ_U830C_ and Qβ_C2011A_.

Preferential selection of mutation U830C over C2011A at 43°C may also be due to the small increase in burst size it produces at this temperature. In all other conditions tested, both C2011A and U830C mutations produce decreases in burst size (Table 2), something for which, with the data we have available, we cannot offer an explanation. However, the increase in burst size would not clarify why mutation U830C is not selected at 43°C at the higher bacterial density assayed. A possible explanation might be the small increase in the latent period that is also caused by this mutation at 43°C.

Additional evolution experiments have shown that Qβ_U830C_ propagated at 37°C (non-selective temperature) at high and low bacterial densities could be maintained at this temperature (Figure 9A). However, at high bacterial densities mutation U830C was selected against, something that did not happen at low bacterial density. In that case the change U830C could be conserved and mutation C2011A was not selected in 10 transfers, which agrees with the non-significant fitness cost of U830C at 37°C and low bacterial density (3 × 10^5^ cfu/ml) (Figure 6). A similar experiment performed with Qβ_C2011A_ propagated at 43°C (non-selective temperature) at low host density showed an increase in the virus titers as the number of transfers increased (Figure 9B). Mutation C2011A was kept, although in the two replicas performed mutation U830C was also selected, indicating that it is a necessary requirement for adaptation to low bacterial density at 43°C. Conversely, lineages propagated at high host density decreased their titers in a progressive way (Figure 9B). Sequencing of the populations obtained at transfer number 10 showed that they had lost mutation C2011A. The results suggest that the negative effect of mutation C2011A at 43°C and high host density reduce virus replication so much that the mutations necessary for adaptation to this temperature cannot be selected. Although the mutation is selected against, probably when this happens it is too late for the virus to increase the titers.

The most important conclusion that can be drawn from this work is that adaptation of Qβ to reduced host availability can be addressed by at least two different mutations that increase virus entry into bacteria. The choice of one or other depends on environmental parameters such as temperature that may affect the strength of the virus-cell interaction, the ease of internalizing the virus genome or the ability to generate an infectious progeny. The balance between the advantages and the fitness costs of each mutation under the particular conditions of virus replication is what decides if it is selected or not.

## Data availability statement

The original contributions presented in this study are included in the article/Supplementary material, further inquiries can be directed to the corresponding author.

## Author contributions

EL designed the study and wrote the manuscript. ML-C, AR-M, and ELl performed the experiments. EL and ML-C analyzed the data. All authors interpreted the results and reviewed the manuscript.

## Footnotes

1
https://www.agilent.com/store/biocalculators/calcODBacterial.jsp

2
https://www.geneious.com

## References

[B1] Abedon S. Herschler T. Stopar D. (2001). Bacteriophage latent-period evolution as a response to resource availability. *Appl. Environ. Microbiol.* 67 4233–4241. 10.1128/AEM.67.9.4233-4241.2001 11526028 PMC93152

[B2] Abedon S. T. (2016). Bacteriophage exploitation of bacterial biofilms: phage preference for less mature targets? *FEMS Microbiol. Lett.* 363:fnv246. 10.1093/femsle/fnv246 26738755

[B3] Abedon S. T. Culler R. R. (2007). Optimizing bacteriophage plaque fecundity. *J. Theor. Biol.* 249 582–592. 10.1016/j.jtbi.2007.08.006 17919662

[B4] Abedon S. T. Hyman P. Thomas C. (2003). Experimental examination of bacteriophage latent-period evolution as a response to bacterial availability. *Appl. Environ. Microbiol.* 69 7499–7506. 10.1128/AEM.69.12.7499-7506.2003 14660403 PMC310036

[B5] Abedon S. T. Yin J. (2009). Bacteriophage plaques: theory and analysis. *Methods Mol. Biol.* 501 161–174. 10.1007/978-1-60327-164-6_17 19066821

[B6] Arribas M. Aguirre J. Manrubia S. Lázaro E. (2018). Differences in adaptive dynamics determine the success of virus variants that propagate together. *Virus Evol.* 4:vex043. 10.1093/ve/vex043 29340211 PMC5761584

[B7] Arribas M. Cabanillas L. Kubota K. Lázaro E. (2016). Impact of increased mutagenesis on adaptation to high temperature in bacteriophage Qβ. *Virology* 497 163–170. 10.1016/j.virol.2016.07.007 27471955

[B8] Arribas M. Kubota K. Cabanillas L. Lázaro E. (2014). Adaptation to fluctuating temperatures in an RNA virus is driven by the most stringent selective pressure. *PLoS One* 9:e100940. 10.1371/journal.pone.0100940 24963780 PMC4071030

[B9] Arribas M. Lázaro E. (2021). Intra-population competition during adaptation to increased temperature in an RNA bacteriophage. *Int. J. Mol. Sci.* 22:6815. 10.3390/ijms22136815 34202838 PMC8268601

[B10] Aviram I. Rabinovitch A. (2008). Dynamical types of bacteria and bacteriophages interaction: shielding by debris. *J. Theor. Biol.* 251 121–136. 10.1016/j.jtbi.2007.11.003 18160076

[B11] Barrera I. Schuppli D. Sogo J. M. Weber H. (1993). Different mechanisms of recognition of bacteriophage Q beta plus and minus strand RNAs by Q beta replicase. *J. Mol. Biol.* 232 512–521. 10.1006/jmbi.1993.1407 8345521

[B12] Bernhardt T. G. Wang I. N. Struck D. K. Young R. (2001). A protein antibiotic in the phage Qβ virion: diversity in lysis targets. *Science* 292 2326–2329. 10.1126/science.1058289 11423662

[B13] Brown E. D. Vivas E. I. Walsh C. T. Kolter R. (1995). MurA (MurZ), the enzyme that catalyzes the first committed step in peptidoglycan biosynthesis, is essential in *Escherichia coli*. *J. Bacteriol.* 177 4194–4197. 10.1128/jb.177.14.4194-4197.1995 7608103 PMC177162

[B14] Bruinsma R. F. Wuite G. J. L. Roos W. H. (2021). Physics of viral dynamics. *Nat. Rev. Phys.* 3 76–91. 10.1038/s42254-020-00267-1 33728406 PMC7802615

[B15] Bull J. J. Gill J. J. (2014). The habits of highly effective phages: population dynamics as a framework for identifying therapeutic phages. *Front. Microbiol.* 5:618. 10.3389/fmicb.2014.00618 25477869 PMC4235362

[B16] Chantranupong L. Heineman R. H. (2012). A common, non-optimal phenotypic endpoint in experimental adaptations of bacteriophage lysis time. *BMC Evol. Biol.* 12:37. 10.1186/1471-2148-12-37 22429718 PMC3324380

[B17] Cui Z. Gorzelnik K. V. Chang J.-Y. Langlais C. Jakana J. Young R. (2017). Structures of Qβ virions, virus-like particles, and the Qβ-MurA complex reveal internal coat proteins and the mechanism of host lysis. *Proc. Natl. Acad. Sci. U.S A.* 114 11697–11702. 10.1073/pnas.1707102114 29078304 PMC5676892

[B18] De Paepe M. Taddei F. (2006). Viruses’ life history: towards a mechanistic basis of a trade-off between survival and reproduction among phages. *PLoS Biol.* 4:e193. 10.1371/journal.pbio.0040193 16756387 PMC1475768

[B19] Dennehy J. J. Abedon S. T. (2020a). “Phage infection and lysis,” in *Bacteriophages*, eds Harper D. R. Abedon S. T. Burrowes B. H. McConville M. L. (Norderstedt: BoD – Books on Demand), 1–43. 10.1007/978-3-319-40598-8_53-1

[B20] Dennehy J. J. Abedon S. T. (2020b). “Bacteriophage ecology,” in *Bacteriophages: biology, technology, therapy*, eds Harper D. R. Abedon S. T. Burrowes B. H. McConville M. L. (Cham: Springer International Publishing), 1–42. 10.1007/978-3-319-40598-8_8-1

[B21] Dennehy J. J. Abedon S. T. Turner P. E. (2007). Host density impacts relative fitness of bacteriophage Φ6 genotypes in structured habitats. *Evolution* 61 2516–2527. 10.1111/j.1558-5646.2007.00205.x 17725627

[B22] Dent K. C. Thompson R. Barker A. M. Hiscox J. A. Barr J. N. Stockley P. G. (2013). The asymmetric structure of an icosahedral virus bound to its receptor suggests a mechanism for genome release. *Structure* 21 1225–1234. 10.1016/j.str.2013.05.012 23810697 PMC3701328

[B23] Elena S. F. (2001). Evolutionary history conditions the timing of transmission in vesicular stomatitis virus. *Infect. Genet. Evol.* 1 151–159. 10.1016/S1567-1348(01)00022-3 12798030

[B24] Farafonov V. S. Nerukh D. (2019). MS2 bacteriophage capsid studied using all-atom molecular dynamics. *Interface Focus* 9:20180081. 10.1098/rsfs.2018.0081 31065345 PMC6501343

[B25] FarafonovV. S.StichM.NerukhD. (2022). Reconstruction and validation of entire virus model with complete genome from mixed resolution cryo-EM density. *Faraday Discuss.* 240 152–167. 10.1039/d2fd00053a 35916040

[B26] Gallet R. Kannoly S. Wang I.-N. (2011). Effects of bacteriophage traits on plaque formation. *BMC Microbiol.* 11:181. 10.1186/1471-2180-11-181 21827665 PMC3176204

[B27] Goldhill D. H. Turner P. E. (2014). The evolution of life history trade-offs in viruses. *Curr. Opin. Virol.* 8 79–84. 10.1016/j.coviro.2014.07.005 25087040

[B28] Gorzelnik K. V. Cui Z. Reed C. A. Jakana J. Young R. Zhang J. (2016). Asymmetric cryo-EM structure of the canonical Allolevivirus Qβ reveals a single maturation protein and the genomic ssRNA in situ. *Proc. Natl. Acad. Sci. U S A.* 113 11519–11524. 10.1073/pnas.1609482113 27671640 PMC5068298

[B29] Gorzelnik K. V. Zhang J. (2021). Cryo-EM reveals infection steps of single-stranded RNA bacteriophages. *Prog. Biophys. Mol. Biol.* 160 76–83. 10.1016/j.pbiomolbio.2020.07.011 32841651

[B30] Harb L. Chamakura K. Khara P. Christie P. J. Young R. Zeng L. (2020). ssRNA phage penetration triggers detachment of the F-pilus. *Proc. Natl. Acad. Sci. U.S.A.* 117 25751–25758. 10.1073/pnas.2011901117 32989140 PMC7568308

[B31] Hayes W. (1953). The mechanism of genetic recombination in *Escherichia coli*. *Cold Spring Harbor Symposia Q. Biol.* 18 75–93. 10.1101/SQB.1953.018.01.016 13168972

[B32] Heineman R. H. Bull J. J. (2007). Testing optimality with experimental evolution: lysis time in a bacteriophage. *Evolution* 61 1695–1709. 10.1111/j.1558-5646.2007.00132.x 17598749 PMC1974807

[B33] Hofstetter H. Monstein H. Weissmann C. (1974). The readthrough protein A1 is essential for the formation of viable Qβ particles. *Biochim. Biophys. Acta* 374 238–251. 4611493 10.1016/0005-2787(74)90366-9

[B34] Hyman P. Abedon S. T. (2009). “Practical methods for determining phage growth parameters,” in *Bacteriophages: Methods in molecular biology^TM^*, Vol. 501 eds Clokie M. R. Kropinski A. M. (Totowa, NJ: Humana Press), 307. 10.1007/978-1-60327-164-6_18 19066822

[B35] Inomata T. Kimura H. Hayasaka H. Shiozaki A. Fujita Y. Kashiwagi A. (2012). Quantitative comparison of the RNA bacteriophage Qβ infection cycle in rich and minimal media. *Arch. Virol.* 157 2163–2169. 10.1007/s00705-012-1419-3 22825697

[B36] Jana A. K. May E. R. (2020). Structural and dynamic asymmetry in icosahedrally symmetric virus capsids. *Curr. Opin. Virol.* 45 8–16. 10.1016/j.coviro.2020.06.002 32615360 PMC7746594

[B37] Karnik S. Billeter M. (1983). The lysis function of RNA bacteriophage Qbeta is mediated by the maturation (A2) protein. *EMBO J.* 2 1521–1526. 11892805 10.1002/j.1460-2075.1983.tb01617.x PMC555316

[B38] Kashiwagi A. Sugawara R. Sano Tsushima F. Kumagai T. Yomo T. (2014). Contribution of silent mutations to thermal adaptation of RNA bacteriophage Qβ. *J. Virol.* 88 11459–11468. 10.1128/JVI.01127-14 25056887 PMC4178783

[B39] Kozak M. Nathans D. (1971). Fate of maturation protein during infection by coliphage MS2. *Nat. New Biol.* 234 209–211. 10.1038/newbio234209a0 4942983

[B40] Krahn P. M. O’Callaghan R. J. Paranchych W. (1972). Stages in phage R17 infection. VI. Injection of a protein and RNA into the host cell. *Virology* 47 628–637. 10.1016/0042-6822(72)90552-1 4551992

[B41] Laguna-Castro M. Lázaro E. (2022). Propagation of an RNA bacteriophage at low host density leads to a more efficient virus entry. *Front. Virol.* 2:858227. 10.3389/fviro.2022.858227

[B42] Lázaro E. Arribas M. Cabanillas L. Román I. Acosta E. (2018). Evolutionary adaptation of an RNA bacteriophage to the simultaneous increase in the within-host and extracellular temperatures. *Sci. Rep.* 8 1–9. 10.1038/s41598-018-26443-z 29795535 PMC5967308

[B43] Manchak J. Anthony K. G. Frost L. S. (2002). Mutational analysis of F-pilin reveals domains for pilus assembly, phage infection and DNA transfer. *Mol. Microbiol.* 43 195–205. 10.1046/j.1365-2958.2002.02731.x 11849547

[B44] Meng R. Jiang M. Cui Z. Chang J.-Y. Yang K. Jakana J. (2019). Structural basis for the adsorption of a single-stranded RNA bacteriophage. *Nat. Commun.* 10:3130. 10.1038/s41467-019-11126-8 31311931 PMC6635492

[B45] Olsthoorn R. C. L. Van Duin J. (2011). *Leviviridae-Positive Sense RNA Viruses-Positive Sense RNA Viruses.* London: ICTV.

[B46] Paranchych W. (1966). Stages in phage R17 infection: the role of divalent cations. *Virology* 28 90–99. 10.1016/0042-6822(66)90309-6 4955195

[B47] Paranchych W. Ainsworth S. K. Dick A. J. Krahn P. M. (1971). Stages in phage R17 infection. V. Phage eclipse and the role of F pili. *Virology* 45 615–628. 10.1016/0042-6822(71)90176-0 4108185

[B48] Rabinovitch A. Aviram I. Zaritsky A. (2003). Bacterial debris-an ecological mechanism for coexistence of bacteria and their viruses. *J. Theor. Biol.* 224 377–383. 10.1016/s0022-5193(03)00174-7 12941595

[B49] Reed C. A. Langlais C. Kuznetsov V. Young R. (2012). Inhibitory mechanism of the Qβ lysis protein A2. *Mol. Microbiol.* 86 836–844. 10.1111/mmi.12021 22934834 PMC4631118

[B50] Reed C. A. Langlais C. Wang I. N. Young R. (2013). A2 expression and assembly regulates lysis in Qβ infections. *Microbiol* 159 507–514. 10.1099/mic.0.064790-0 23329676 PMC3709820

[B51] Reynolds S. Paranchych W. (1976). The isolation of an infectious A protein RNA complex from coliphage R17. *Can. J. Microbiol.* 22 1647–1653. 10.1139/m76-242 974912

[B52] Rumnieks J. Tars K. (2011). Crystal structure of the read-through domain from bacteriophage Qβ A1 protein. *Protein Sci.* 20 1707–1712. 10.1002/pro.704 21805520 PMC3218364

[B53] Rumnieks J. Tars K. (2017). Crystal structure of the maturation protein from bacteriophage Qβ. *J. Mol. Biol.* 429 688–696. 10.1016/j.jmb.2017.01.012 28111107

[B54] Shao Y. Wang I.-N. (2008). Bacteriophage adsorption rate and optimal lysis time. *Genetics* 180 471–482. 10.1534/genetics.108.090100 18757924 PMC2535697

[B55] Shiba T. Miyake T. (1975). New type of infectious complex of E. coli RNA phage. *Nature* 254 157–158. 10.1038/254157a0 1090846

[B56] Somovilla P. Manrubia S. Lázaro E. (2019). Evolutionary dynamics in the RNA bacteriophage Qβ depends on the pattern of change in selective pressures. *Pathog* 8:80. 10.3390/pathogens8020080 31216651 PMC6631425

[B57] Somovilla P. Rodríguez-Moreno A. Arribas M. Manrubia S. Lázaro E. (2022). Standing genetic diversity and transmission bottleneck size drive adaptation in bacteriophage Qβ. *Int. J. Mol. Sci.* 23:8876. 10.3390/ijms23168876 36012143 PMC9408265

[B58] Taniguchi T. Palmieri M. Weissmann C. (1978). QB DNA-containing hybrid plasmids giving rise to QB phage formation in the bacterial host. *Nature* 274 223–228. 10.1038/274223a0 355887

[B59] Toropova K. Stockley P. G. Ranson N. A. (2011). Visualising a viral RNA genome poised for release from its receptor complex. *J. Mol. Biol.* 408 408–419. 10.1016/j.jmb.2011.02.040 21376055

[B60] Valentine R. C. Strand M. (1965). Complexes of F-pili and RNA bacteriophage. *Science* 148 511–513. 10.1126/science.148.3669.511 14263773

[B61] Vasiljeva I. Kozlovska T. Cielens I. Strelnikova A. Kazaks A. Ose V. (1998). Mosaic Qbeta coats as a new presentation model. *FEBS Lett.* 431 7–11. 10.1016/s0014-5793(98)00716-9 9684855

[B62] Wasik B. R. Bhushan A. Ogbunugafor C. B. Turner P. E. (2015). Delayed transmission selects for increased survival of vesicular stomatitis virus. *Evolution* 69 117–125. 10.1111/evo.12544 25311513

[B63] Winter R. B. Gold L. (1983). Overproduction of bacteriophage Qβ maturation (A2) protein leads to cell lysis. *Cell* 33 877–885. 10.1016/0092-8674(83)90030-2 6871998

[B64] Woody M. A. Cliver D. O. (1995). Effects of temperature and host cell growth phase on replication of F- specific RNA coliphage Qβ. *Appl. Environ. Microbiol.* 61 1520–1526. 10.1128/aem.61.4.1520-1526.1995 7747969 PMC167408

[B65] Yin J. Redovich J. (2018). Kinetic modeling of virus growth in cells. *Microbiol. Mol. Biol. Rev.* 82 1–33. 10.1128/mmbr.00066-17 29592895 PMC5968458

