# Supplementary material for: The balance between fitness advantages and costs drives adaptation of bacteriophage Qβ to changes in host density at different temperatures
Source: Front Microbiol. 2023 May 25;14:1197085. doi: 10.3389/fmicb.2023.1197085 (PMC10248866; doi:10.3389/fmicb.2023.1197085)
Supplement: Supplementary file 1 [file Data_Sheet_1.pdf]

Supplementary Table 1. Raw data corresponding to Figure 3A

Bacterial density (cfu/mL)	Virus population assayed[1]	Virus titers (pfu/mL)[2]	Mean ± SD[3]
3×10^8	$Q\beta_{Anc}$	2.8×10^7<br>2.1×10^7<br>3.6×10^7	$2.8 \times 10^7 \pm 7.5 \times 10^6$
3×10^8	$Q\beta(3\times10^8)_{30°C}$ (1)	8.0×10^7<br>7.0×10^7<br>6.0×10^7	$7.0 \times 10^7 \pm 1.0 \times 10^7$
3×10^8	$Q\beta(3\times10^8)_{30°C}$ (2)	9.0×10^7<br>1.2×10^8<br>1.2×10^8	$1.1 \times 10^8 \pm 1.7 \times 10^7$
3×10^7	$Q\beta_{Anc}$	5.4×10^8<br>4.3×10^8<br>7.9×10^8	$5.9 \times 10^8 \pm 1.8 \times 10^8$
3×10^7	$Q\beta(3\times10^7)_{30°C}$ (1)	3.7×10^9<br>3.5×10^7<br>3.8×10^9	$2.5 \times 10^9 \pm 2.1 \times 10^9$
3×10^7	$Q\beta(3\times10^7)_{30°C}$ (2)	4.9×10^9<br>6.1×10^9<br>5.2×10^9	$5.4 \times 10^9 \pm 6.2 \times 10^8$
3×10^6	$Q\beta_{Anc}$	8.0×10^6<br>1.5×10^7<br>1.7×10^7	$1.3 \times 10^7 \pm 4.6 \times 10^6$
3×10^6	$Q\beta(3\times10^6)_{30°C}$ (1)	1.9×10^8<br>2.0×10^8<br>1.7×10^8	$1.9 \times 10^8 \pm 1.7 \times 10^7$
3×10^6	$Q\beta(3\times10^6)_{30°C}$ (2)	5.6×10^7<br>7.3×10^7<br>7.5×10^7	$6.8 \times 10^7 \pm 1.0 \times 10^7$

[1](1) and (2) refer to the two replicate lineages evolved in parallel (see Figure 1).

[2]The values indicated correspond to the three replicas performed for each condition.

[3]Mean and standard deviation of the three data points obtained for each condition.

Supplementary Table 2. Raw data corresponding to Figure 3B

Bacterial density (cfu/mL)	Virus population assayed[1]	Virus titers (pfu/mL)[2]	Mean ± SD[3]
3×10^8	$Q\beta_{Anc}$	1.4×10^7<br>8.0×10^6<br>8.0×10^6	$1.0 \times 10^7 \pm 3.5 \times 10^6$
3×10^8	$Q\beta(3{\times}10^8)_{43°C}$ (1)	5.2×10^8<br>3.7×10^8<br>4.1×10^8	$4.3 \times 10^8 \pm 7.8 \times 10^7$
3×10^8	$Q\beta(3{\times}10^8)_{43°C}$ (2)	2.8×10^8<br>3.2×10^8<br>2.9×10^8	$3.0 \times 10^8 \pm 2.0 \times 10^7$
3×10^7	$Q\beta_{Anc}$	1.4×10^7<br>1.5×10^7<br>2.0×10^7	$1.6 \times 10^7 \pm 3.2 \times 10^6$
3×10^7	$Q\beta(3{\times}10^7)_{43°C}$ (1)	8.0×10^8<br>4.5×10^8<br>4.6×10^8	$5.8 \times 10^8 \pm 2.2 \times 10^8$
3×10^7	$Q\beta(3{\times}10^7)_{43°C}$ (2)	2.8×10^8<br>4.3×10^8<br>2.0×10^8	$3.0 \times 10^8 \pm 6.2 \times 10^8$
3×10^6	$Q\beta_{Anc}$	5.6×10^6<br>7.1×10^6<br>6.7×10^6	$6.5 \times 10^6 \pm 1.2 \times 10^5$
3×10^6	$Q\beta(3{\times}10^6)_{43°C}$ (1)	1.8×10^9<br>2.1×10^9<br>2.3×10^9	$2.1 \times 10^9 \pm 2.3 \times 10^8$
3×10^6	$Q\beta(3{\times}10^6)_{43°C}$ (2)	1.5×10^9<br>2.3×10^9<br>1.8×10^9	$1.9 \times 10^9 \pm 3.7 \times 10^8$

[1](1) and (2) refer to the two replicate lineages evolved in parallel (see Figure 1).

[2]The values indicated correspond to the three replicas performed for each condition.

[3]Mean and standard deviation of the three data points obtained for each condition.

Supplementary Table 3. Raw data corresponding to Figure 5A

Bacterial density (cfu/mL)	Virus assayed	Virus titers[1] (pfu/mL)	Mean ± SD[2]
3×10^8	$Q\beta_{Anc}$	7.0×10^7<br>6.1×10^7<br>8.3×10^7	$7.1 \times 10^7 \pm 1.1 \times 10^7$
3×10^8	$Q\beta_{C2011A}$	2.6×10^8<br>2.1×10^8<br>1.5×10^8	$2.1 \times 10^8 \pm 5.5 \times 10^7$
3×10^7	$Q\beta_{Anc}$	7.6×10^8<br>7.1×10^8<br>6.3×10^8	$7.0 \times 10^8 \pm 6.6 \times 10^7$
3×10^7	$Q\beta_{C2011A}$	3.2×10^9<br>4.5×10^9<br>4.5×10^9	$4.1 \times 10^9 \pm 7.5 \times 10^8$
3×10^6	$Q\beta_{Anc}$	5.3×10^6<br>1.1×10^7<br>6.6×10^6	$7.8 \times 10^6 \pm 3.2 \times 10^6$
3×10^6	$Q\beta_{C2011A}$	8.6×10^7<br>5.0×10^7<br>8.0×10^7	$7.2 \times 10^7 \pm 1.9 \times 10^7$

[1]The values indicated correspond to the three replicas performed for each condition.

[2]Mean and standard deviation of the three data points obtained for each condition.

Supplementary Table 4. Raw data corresponding to Figure 5B

Bacterial density (cfu/mL)	Virus assayed	Virus titers[1] (pfu/mL)	Mean ± SD[2]
3×10^8	$Q\beta_{Anc}$	1.2×10^7<br>3.3×10^7<br>4.1×10^7	$2.9 \times 10^7 \pm 1.5 \times 10^7$
3×10^8	$Q\beta_{U830C}$	6.0×10^6<br>5.4×10^6<br>3.6×10^6	$5.0 \times 10^6 \pm 1.3 \times 10^6$
3×10^7	$Q\beta_{Anc}$	9.4×10^7<br>9.0×10^7<br>1.1×10^8	$9.8 \times 10^7 \pm 1.1 \times 10^7$
3×10^7	$Q\beta_{U830C}$	2.5×10^8<br>3.1×10^8<br>3.8×10^8	$3.1 \times 10^8 \pm 6.2 \times 10^7$
3×10^6	$Q\beta_{Anc}$	6.5×10^6<br>9.6×10^6<br>7.5×10^6	$7.9 \times 10^6 \pm 1.6 \times 10^6$
3×10^6	$Q\beta_{U830C}$	2.6×10^7<br>3.0×10^7<br>5.3×10^7	$3.6 \times 10^7 \pm 1.5 \times 10^7$

[1]The values indicated correspond to the three replicas performed for each condition.

[2]Mean and standard deviation of the three data points obtained for each condition.

Supplementary Table 5. Raw data corresponding to Figure 6A

Bacterial density (cfu/mL)	Virus assayed	Virus titers[1] (pfu/mL)	Mean ± SD[2]
3×10^8	$Q\beta_{Anc}$	1.3×10^7<br>3.2×10^7<br>2.7×10^7	$2.4 \times 10^7 \pm 9.9 \times 10^6$
3×10^8	$Q\beta_{U830C}$	8.0×10^5<br>6.0×10^5<br>1.3×10^6	$9.0 \times 10^5 \pm 3.6 \times 10^5$
3×10^7	$Q\beta_{Anc}$	1.1×10^8<br>1.1×10^8<br>7.2×10^7	$9.5 \times 10^7 \pm 2.0 \times 10^7$
3×10^7	$Q\beta_{U830C}$	2.7×10^6<br>3.0×10^6<br>3.5×10^6	$3.1 \times 10^6 \pm 4.0 \times 10^5$
3×10^6	$Q\beta_{Anc}$	1.8×10^6<br>2.3×10^6<br>1.6×10^6	$1.9 \times 10^6 \pm 3.6 \times 10^5$
3×10^6	$Q\beta_{U830C}$	5.4×10^5<br>6.0×10^5<br>6.8×10^5	$6.1 \times 10^5 \pm 7.0 \times 10^4$

[1]The values indicated correspond to the three replicas performed for each condition.

[2]Mean and standard deviation of the three data points obtained for each condition.

Supplementary Table 6. Raw data corresponding to Figure 6B

Bacterial density (cfu/mL)	Virus assayed	Virus titers[1] (pfu/mL)	Mean ± SD[2]
3×10^8	$Q\beta_{Anc}$	3.8×10^9<br>1.8×10^9<br>2.4×10^9	$2.7 \times 10^9 \pm 1.0 \times 10^9$
3×10^8	$Q\beta_{U830C}$	2.8×10^7<br>1.7×10^7<br>1.0×10^7	$1.8 \times 10^7 \pm 9.1 \times 10^6$
3×10^7	$Q\beta_{Anc}$	1.0×10^{11}<br>1.2×10^{11}<br>9.0×10^{10}	$1.0 \times 10^{11} \pm 1.5 \times 10^{10}$
3×10^7	$Q\beta_{U830C}$	1.4×10^9<br>4.2×10^9<br>3.4×10^9	$3.0 \times 10^9 \pm 1.4 \times 10^9$
3×10^6	$Q\beta_{Anc}$	2.4×10^{10}<br>1.4×10^{10}<br>2.0×10^{10}	$1.9 \times 10^{10} \pm 5.0 \times 10^9$
3×10^6	$Q\beta_{U830C}$	5.1×10^8<br>5.7×10^8<br>8.8×10^8	$6.5 \times 10^8 \pm 2.0 \times 10^8$
3×10^5	$Q\beta_{Anc}$	3.2×10^7<br>4.5×10^7<br>6.1×10^7	$4.6 \times 10^7 \pm 1.5 \times 10^7$
3×10^5	$Q\beta_{U830C}$	1.1×10^7<br>1.4×10^7<br>1.8×10^7	$1.4 \times 10^7 \pm 3.6 \times 10^6$

[1]The values indicated correspond to the three replicas performed for each condition.

[2]Mean and standard deviation of the three data points obtained for each condition.

Supplementary Table 7. Raw data corresponding to Figure 6C

Bacterial density (cfu/mL)	Virus assayed	Virus titers[1] (pfu/mL)	Mean ± SD[2]
3×10^8	$Q\beta_{Anc}$	1.4×10^8<br>1.4×10^8<br>1.2×10^8	$1.3 \times 10^8 \pm 1.3 \times 10^7$
3×10^8	$Q\beta_{C2011A}$	1.9×10^7<br>1.8×10^7<br>1.8×10^7	$1.8 \times 10^7 \pm 3.5 \times 10^5$
3×10^7	$Q\beta_{Anc}$	3.0×10^8<br>2.9×10^8<br>1.2×10^8	$2.3 \times 10^8 \pm 9.8 \times 10^7$
3×10^7	$Q\beta_{C2011A}$	2.0×10^7<br>1.8×10^7<br>1.9×10^7	$1.9 \times 10^7 \pm 8.2 \times 10^5$
3×10^6	$Q\beta_{Anc}$	1.6×10^7<br>1.1×10^7<br>1.4×10^7	$1.4 \times 10^7 \pm 2.4 \times 10^6$
3×10^6	$Q\beta_{C2011A}$	1.0×10^7<br>1.1×10^7<br>6.2×10^6	$9 \times 10^6 \pm 2.4 \times 10^6$

[1]The values indicated correspond to the three replicas performed for each condition.

[2]Mean and standard deviation of the three data points obtained for each condition.

Supplementary Table 8. Raw data corresponding to Figure 8

Panel A (30 °C)

Virus assayed	Virus titers[1] (pfu/mL) (NB)	Mean ± SD[2] (NB)	Virus titers[1] (pfu/mL) (lysate)	Mean ± SD[2] (lysate)
Qβ_{Anc}	1.9×10^5<br>2.1×10^5<br>2.2×10^5	$2.1 \times 10^5 \pm 1.5 \times 10^4$	7.1×10^4<br>7.0×10^4<br>6.5×10^4	$6.9 \times 10^4 \pm 3.2 \times 10^3$
Qβ_{U830C}	1.7×10^5<br>1.8×10^5<br>2.3×10^5	$1.9 \times 10^5 \pm 3.2 \times 10^4$	6.0×10^4<br>5.6×10^4<br>5.9×10^4	$5.8 \times 10^4 \pm 2.1 \times 10^3$
Qβ_{C2011A}	2.5×10^5<br>2.0×10^5<br>1.8×10^5	$2.1 \times 10^5 \pm 3.6 \times 10^4$	6.6×10^4<br>6.3×10^4<br>6.6×10^4	$6.5 \times 10^4 \pm 1.7 \times 10^3$

Panel B (37 °C)

Virus assayed	Virus titers[1] (pfu/mL) (NB)	Mean ± SD[2] (NB)	Virus titers[1] (pfu/mL) (lysate)	Mean ± SD[2] (lysate)
Qβ_{Anc}	2.9×10^5<br>2.8×10^5<br>2.3×10^5	$2.7 \times 10^5 \pm 3.2 \times 10^4$	2.4×10^4<br>2.0×10^4<br>1.9×10^4	$2.1 \times 10^4 \pm 2.7 \times 10^3$
Qβ_{U830C}	2.3×10^5<br>2.2×10^5<br>2.6×10^5	$2.4 \times 10^5 \pm 2.1 \times 10^4$	2.0×10^4<br>2.6×10^4<br>2.5×10^4	$2.4 \times 10^4 \pm 3.2 \times 10^3$
Qβ_{C2011A}	2.6×10^5<br>2.3×10^5<br>2.5×10^5	$2.5 \times 10^5 \pm 1.5 \times 10^4$	2.2×10^4<br>2.3×10^4<br>1.7×10^4	$2.1 \times 10^4 \pm 3.2 \times 10^3$

Panel C (43 °C)

Virus assayed	Virus titers[1] (pfu/mL) (NB)	Mean ± SD[2] (NB)	Virus titers[1] (pfu/mL) (lysate)	Mean ± SD[2] (lysate)
Qβ_{Anc}	1.1×10^5<br>1.4×10^5<br>1.6×10^5	$1.4 \times 10^5 \pm 2.5 \times 10^4$	1.3×10^2<br>1.2×10^2<br>9.0×10^1	$1.1 \times 10^2 \pm 2.1 \times 10^1$
Qβ_{U830C}	1.0×10^5<br>1.5×10^5<br>1.9×10^5	$1.5 \times 10^5 \pm 4.5 \times 10^4$	1.1×10^2<br>1.5×10^2<br>1.6×10^2	$1.4 \times 10^2 \pm 2.7 \times 10^1$
Qβ_{C2011A}	1.5×10^5<br>1.2×10^5<br>1.8×10^5	$1.5 \times 10^5 \pm 3.0 \times 10^4$	1.2×10^1<br>1.6×10^1<br>1.6×10^1	$1.5 \times 10^1 \pm 2.3 \times 10^0$

[1]The values indicated correspond to the three replicas performed for each condition.

[2]Mean and standard deviation of the three data points obtained for each condition.

Supplementary Figure 1. Growth curves of *Escherichia coli* Hfr at 30 °C, 37 °C, and 43 °C

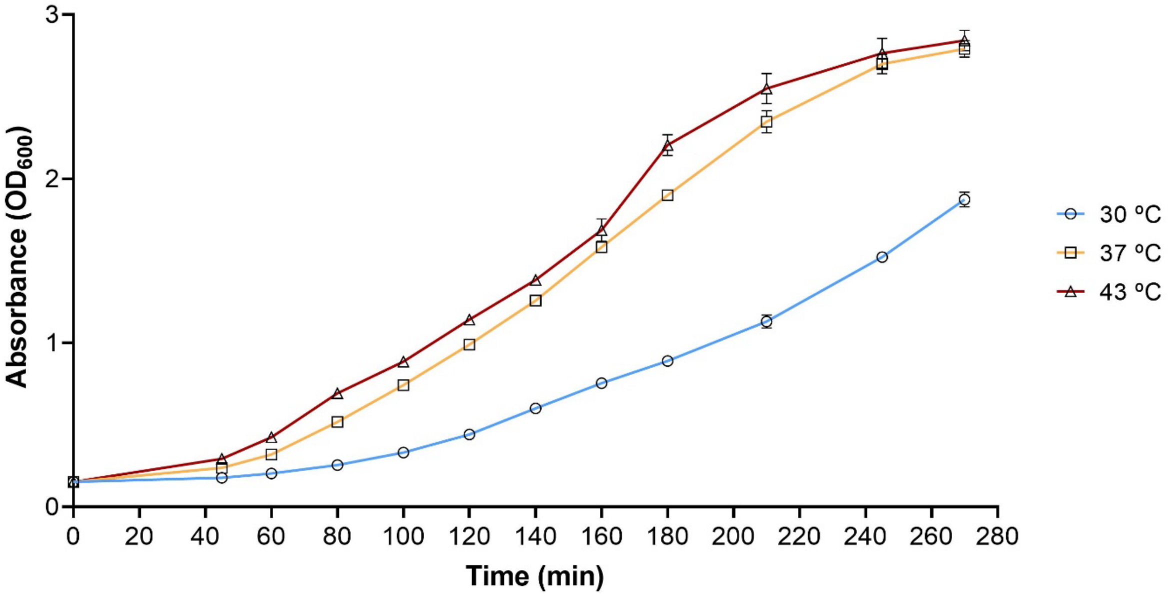

A stationary phase *E. coli* culture was diluted 20-fold in NB medium, in flasks containing a final volume of 20 mL (two flasks per temperature tested). The cultures were incubated at the indicated temperature with shaking (250 rpm). The bacterial density was determined by measuring the optical density at 600 nm.

Supplementary Figure 2. Number of infectious centers produced upon a 10 min incubation of viruses $Q\beta_{Anc}$, $Q\beta_{U830C}$ and $Q\beta_{C2011A}$ at the temperatures indicated

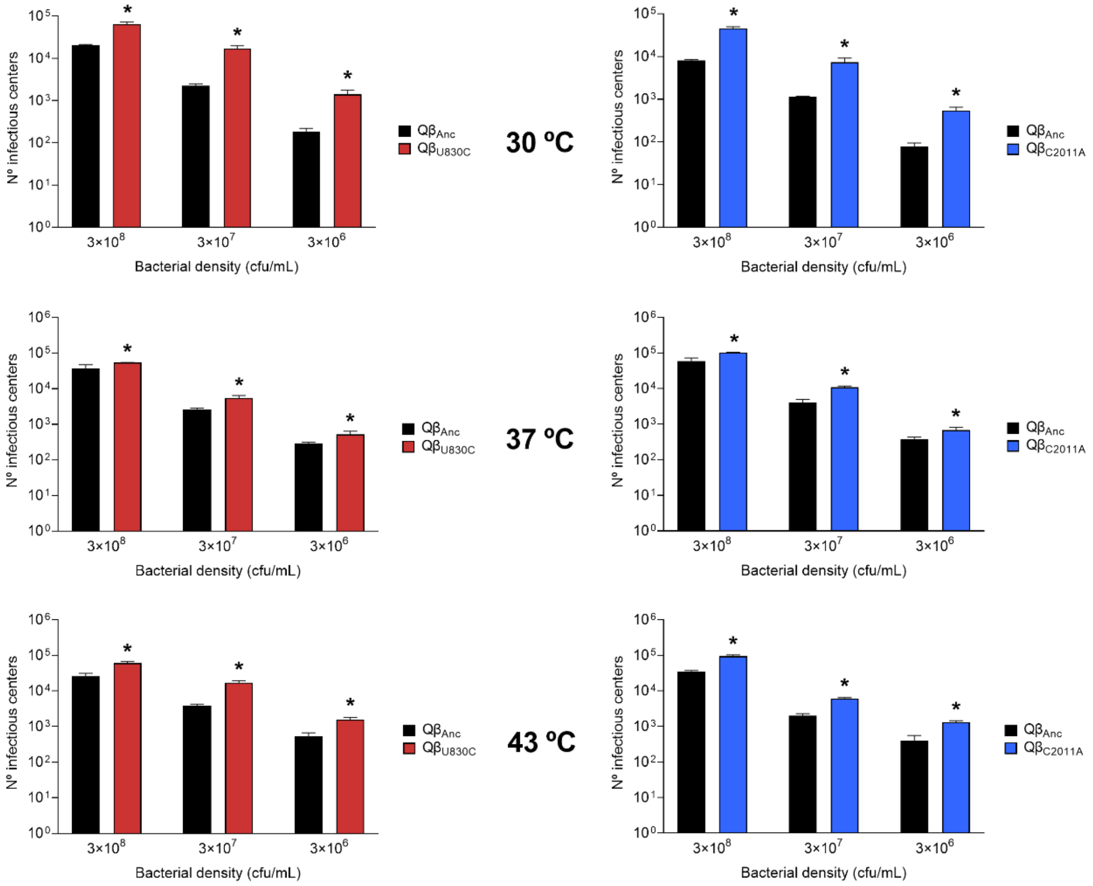

10^5 pfu of either the virus $Q\beta_{Anc}$ (black bars), $Q\beta_{U830C}$ (red bars), or $Q\beta_{C2011A}$ (blue bars) were incubated with different concentrations of bacteria (indicated at the bottom of the figure) for 10 min, after which the number of infectious centers was determined (see section Determination of virus entry into bacteria in Materials and Methods). Each bar represents the average of three determinations. Asterisks above the bars indicate that the result is significantly different from the value obtained for the virus $Q\beta_{Anc}$ ($p < 0.05$) according to a Student *t* test.
